# Squalenoyl siRNA PMP22 nanoparticles are effective in treating mouse models of Charcot-Marie-Tooth disease type 1 A

**DOI:** 10.1038/s42003-021-01839-2

**Published:** 2021-03-09

**Authors:** Suzan Boutary, Marie Caillaud, Mévidette El Madani, Jean-Michel Vallat, Julien Loisel-Duwattez, Alice Rouyer, Laurence Richard, Céline Gracia, Giorgia Urbinati, Didier Desmaële, Andoni Echaniz-Laguna, David Adams, Patrick Couvreur, Michael Schumacher, Charbel Massaad, Liliane Massaad-Massade

**Affiliations:** 1 U1195 Diseases and Hormones of the Nervous System, Inserm and University Paris-Saclay, 94276 Le Kremlin-Bicêtre, France; 2 National Research Centre, Cairo, Egypt; 3 Service de Neurologie - Centre de Référence Neuropathies Périphérique Rares, CHU de Limoges - Hôpital Dupuytren, 2 Avenue Martin Luther King, 87042 LIMOGES CEDEX, France; 4 Neurology Department, AP-HP, Université Paris-Saclay and French Reference Center for Familial Amyloid Polyneuropathy and other rare peripheral neuropathies (CRMR-NNERF), Bicêtre University Hospital, Le Kremlin-Bicêtre, France; 5 UMR 8203 CNRS, newly UMR 9018 CNRS, Université Paris-Saclay, 94805 Villejuif, France; 6 Institut Galien Paris-Sud, CNRS UMR 8612, Université Paris-Sud, Université Paris-Saclay, 92290 Châtenay-Malabry, France; 7 Faculty of Basic and Biomedical Sciences, Paris Descartes University, INSERM UMRS 1124, 75006 Paris, France

**Keywords:** Neurodegenerative diseases, Preclinical research

## Abstract

Charcot-Marie-Tooth disease type 1 A (CMT1A) lacks an effective treatment. We provide a therapy for CMT1A, based on siRNA conjugated to squalene nanoparticles (siRNA PMP22-SQ NPs). Their administration resulted in normalization of Pmp22 protein levels, restored locomotor activity and electrophysiological parameters in two transgenic CMT1A mouse models with different severity of the disease. Pathological studies demonstrated the regeneration of myelinated axons and myelin compaction, one major step in restoring function of myelin sheaths. The normalization of sciatic nerve Krox20, Sox10 and neurofilament levels reflected the regeneration of both myelin and axons. Importantly, the positive effects of siRNA PMP22-SQ NPs lasted for three weeks, and their renewed administration resulted in full functional recovery. Beyond CMT1A, our findings can be considered as a potent therapeutic strategy for inherited peripheral neuropathies. They provide the proof of concept for a new precision medicine based on the normalization of disease gene expression by siRNA.

## Abstract (web summary)

Boutary et al. describe siRNA based therapy conjugated with squalene nanoparticles as an efficient approach to normalize PMP22 protein levels, restore locomotor activity, electrophysiological parameters and function of myelin sheath in CMT1A mouse models. These findings could be useful to develop therapeutic strategies for inherited peripheral neuropathies.

## Introduction

Charcot-Marie-Tooth (CMT) diseases are the group of inherited neuropathies caused by chromosomal rearrangements and mutations^1,2^. Demyelinating CMT1A occurs in the first and second decade of life and represents around 40–60% of all CMT cases^3,4^. It is caused by a duplication in chromosome 17p11.2, leading to the overexpression of Pmp22, a 22-kDa hydrophobic transmembrane protein produced by Schwann cells and representing 2–5% of peripheral myelin proteins^1^. Electrophysiological studies demonstrate reduced nerve conduction velocity (NCV) (below 38 m s^−1^) and decreased compound muscle action potential (CMAP)^5^. Histopathological studies usually show onion bulbs, resulting from repetitive episodes of axon demyelination followed by remyelination^6^.

To date, there has been no effective treatment for CMT1A^7^. Existing therapies aim to reduce its progression through rehabilitation and surgical corrections. Most of the therapeutic interventions include modulators of adenylate cyclase activity, such as ascorbic acid^8^ and combination therapy PXT 3003^9^, neurotrophin-3^10^, an antagonist of the progesterone receptor (Onapristone)^11^ and pain-modulating drugs (ADX71441^12^ and FLX-787^13^). However, these molecules did not reach clinics due to their inefficiency or toxicity in clinical trials^7^. More recently, genetic therapy was introduced by Zhao et al. by using antisense oligonucleotides (ASO) in CMT1A animal models and showed promising results^14^.

Here, we took on the challenge of using siRNA to reverse CMT1A disease phenotypes in two transgenic mouse models. Due to their mechanism of action, siRNA offer important advantages, in particular their high degree of safety as they inhibit gene expression at a posttranslational level and do not directly interact with DNA, their high efficacy in suppressing gene expression and their specificity determined by complementary base pairing^15^.

However, although inhibiting the expression of a culprit disease gene by siRNA has been successfully demonstrated, the normalization of an overexpressed dosage-sensitive gene, such as *PMP22* in CMT1A, has never been considered. Such therapeutic strategy faces multiple challenges, in particular the requirement for reaching normal levels of Pmp22, as either too high or too low levels of the protein result in peripheral neuropathy. Whereas overexpression of Pmp22 causes CMT1A, its inhibition below normal levels results in hereditary neuropathy with liability to pressure palsies (HNPP)^16^.

Due to their hydrophilicity and short plasmatic half-life, a major obstacle for siRNA therapy is their delivery to target cells. To overcome these limitations, several viral and non-viral vectors were developed. Viral vectors may show cyto- and geno-toxic adverse effects and are rather difficult to obtain, which limits their clinical applicability^17^. Concerning non-viral vectors, encapsulation using cationic lipids and polymers has been successful in increasing the efficacy and safety of siRNA therapeutics^18^. This was highlighted by the FDA approval of first siRNA “Patisiran” for the treatment of transthyretin-mediated amyloidosis disease. Currently the actual tendency is oriented toward chemical conjugation of siRNA to different molecules^15^. A major advance has been the discovery that bioconjugates resulting from the chemical linkage of small molecules to squalene (SQ), a natural and biocompatible lipid, could self-assemble as nanoparticles (NPs), offering protection and improving pharmacological efficacy of drugs for the treatment of variety of diseases, including cancer, neurological disorders and pain alleviation^19–21^. Noteworthy, the synthesis and preparation of the drug-SQ NPs is easy and represents a flexible platform for drug delivery (for detailed information review^15^). This technology has already been successfully applied to siRNA for silencing oncogenes in prostate cancer and thyroid papillary carcinoma^22–24^.

Here, we show that in preclinical models of CMT1A, the dosed administration of siRNA PMP22-SQ NPs normalizes Pmp22 levels, improves motor and neuromuscular activities, restores electrophysiological endpoints and triggers the remyelination and regeneration of axons. These results open a new avenue for the use of siRNA in the treatment of CMT1A and other diseases caused by unbalanced chromosomal rearrangements and gene copy-number variations.

## Results

### Construction of siRNAs PMP22 and efficacy testing in MSC80 cells

To initiate this study, the common mRNA PMP22 sequences of *homo sapiens* (Supplementary Fig. 1a) and *mus musculus* (Supplementary Fig. 1b), were determined by BLASTN (Supplementary Table 1a). Eight different siRNAs against PMP22 were designed according to Tafer and Reynolds method (Supplementary Table 1b)^25^. To investigate their inhibitory effect on PMP22, siRNAs PMP22 (named: siPMP1, siPMP2, siPMP3, siPMP4, siPMP5, siPMP6, siPMP7, siPMP8) and siRNA control (siRNA Ct), a commercial scramble sequence presenting no homology with any known eukaryotic gene, were transfected into MSC80 mouse Schwann cells. An optimal siRNA candidate should inhibit between 30 and 50% of *PMP22* expression and restore normal levels of Pmp22 protein, which are increased by 1.5- to 2-folds in CMT1A patients as mention by Svaren et al.^26^. In addition, the selected siRNA should exert long-lasting effects, not affecting the expression of myelin protein zero *P0*, which ensures cohesion between the spinal turns of the Schwann cell plasma membrane during myelin formation^27^. Notably, an inhibition greater than 70% of *PMP22* could be expected to result in the development of HNPP^28^. Concerning *P0*, a modification of its expression was described to trigger the development of CMT1B^27,29^. Of the eight siRNAs PMP22 tested, siPMP7 met all the above-listed criteria: it inhibited PMP22 gene expression and protein levels in a long-lasting manner by about 50% without affecting P0 expression (Supplementary Fig. 2a–d). This siRNA PMP22 targeted a region close to the 3′-UTR of PMP22 mRNA. Since the untranslated region is generally conserved after transcription, this could favor an efficient inhibition of the gene expression. Then, we selected the optimal concentration of siPMP7 (50 nM) that showed no significant effect on *P0* and on cell viability, and this condition was used for further experiments and is referred to as siRNA PMP22 for the rest of the study (Supplementary Fig. 2e).

### Naked siRNA PMP22 is not efficient in inhibiting PMP22 in vivo

To check if siRNA PMP22 works in vivo in the absence of nanoparticle protection, we administered 2.5 mg/kg of siRNA PMP22 divided into five intravenous (*i.v.*) injection of 0.5 mg/kg each twice per week to transgenic JP18 mice that carry one extra copy of the *PMP22* gene. Results showed no significant differences between siRNA PMP22 treated and untreated mice for locomotion and muscular strength (Supplementary Fig. 3a and Video 1). Moreover, molecular analysis revealed no inhibition of Pmp22 protein expression (Supplementary Fig. 3b).

### Synthesis of siRNA Control-SQ (siRNA Ct-SQ) and siRNA PMP22-SQ nanoparticle (NPs) resulted in hydrophobic and stable NPs

As the naked siRNA PMP22 had no effect in vivo, we decided to conjugate it to SQ, a safe and biocompatible endogenous triterpene with the ability to form NPs in H_2_O. We conjugated both siRNA PMP22 and siRNA Ct to SQ through a covalent link taking advantage of the “copper-free Click Chemistry”^30^. To this aim, the sense strand was modified by a dibenzocyclooctyne (DBCO) residue at the 5′-end of the siRNA. To avoid steric hindrance, a C6 linker was used. SQ was modified by a terminal azide group (SQ-N3) to react with the DBCO residue of the sense strand siRNAs. A *quasi* quantitative yield of siRNA PMP22-SQ and siRNA control (Ct)-SQ was obtained during the bioconjugation step.

The bioconjugate siRNAs-SQ were more hydrophobic (Supplementary Fig. 4a) and showed a major peak (see MALDI-TOF MS spectrum in Supplementary Fig. 4b). The resulting NPs were stable over the period of 1 month at 4 °C (Supplementary Fig. 4c). They were spherical in shape with a mean diameter of about 180 nm (Supplementary Fig. 4c, d) and a polydispersity index (PDI) of 0.2 ± 0.02 for siRNA PMP22-SQ NP and, with a mean diameter of 255 nm and a PDI of 0.15 ± 0.02 for siRNA Ct-SQ NPs (Supplementary Fig. 4c). Importantly, SQ conjugation did not affect siRNA efficacy. Indeed, siRNA PMP22-SQ NPs still downregulated PMP22 mRNA expression in MSC80 cells similarly to the naked siRNA PMP22 until 72 h without affecting *P0* gene expression and cell viability (Supplementary Fig. 5a, b). These results were in accordance with other studies showing that chemical modifications of siRNA improved their stability without affecting their performance^30,31^. Thus, siRNA squalenoylation by copper-free click chemistry could be used as a platform for siRNA delivery. The synthesized siRNA PMP22-SQ NPs were active in vitro without displaying cytotoxicity.

### JP18 and JP18/JY13 are representative models of CMT1A pathology

**Fig. 1:**
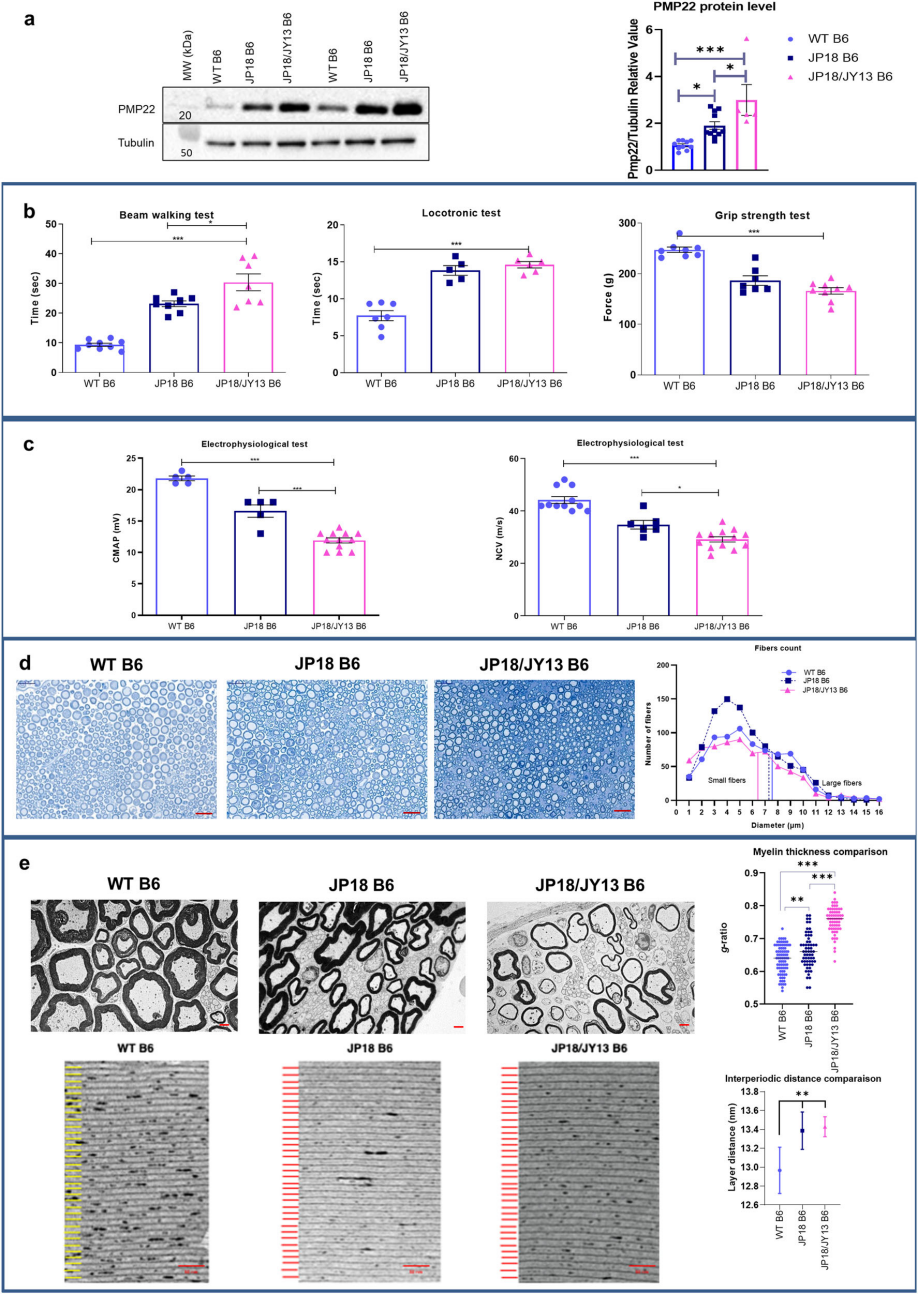
JP18 B6 and JP18/JY13 B6 mice are representative models for CMT1A pathology. **a** Representative western blot gel showing the Pmp22 protein content in sciatic nerve normalized to tubulin then, reported on WT and the corresponding protein quantification. **b** Time taken by the mice to perform the beam walking, the locotronic test and the force of their total limbs in gram. **c** Analysis of the electrophysiological tests CMAP and NCV. **d** Semi-thin section of sciatic nerves from WT B6, JP18 B6, and JP18/JY13 B6 mice scanned at 40×. Scale bar 20 µm. Fiber count quantification was done using image J software at 70×. To determine the cutoff between small and large fibers a quadratic model analysis was performed. The cutoff is: 7.4 with a 95% CI at [6.95–7.85] for WT (blue line), 7.20 CI [6.39–7.99] for JP18 (dark dashed) and 6.35 CI [5.54–7.10] for JP18/JY13 (pink line). **e** Transmission electron microscopy (TEM) images (5kx) of ultrathin sciatic nerve sections of the tested mice groups and the g-ratios analysis of myelinated fibers. Scale bar 2 µm. High magnification images (120kx) were taken to show the inter myelin layer distance which is represented by yellow lines for WT and red lines for JP18 and JP18/JY13 group followed by interperiodic analysis graph. Scale bar 50 nm. All the experiments were done on six mice per group. Data represent predicted means with 95% confidence intervals for layer distance while mean ± s.e.m. was represented elsewhere. *p < 0.05; **p < 0.01; ***p < 0.001 using ANOVA analysis followed by Tukey’s multiple comparison tests.

After in vitro validation of siRNA PMP22-SQ NPs, their therapeutic efficacy was tested in two transgenic mouse models of CMT1A, named JP18 and JP18/JY13, carrying respectively one and two extra copies of the *PMP22* gene and developed on a B6 background^32^. Since birth, the *PMP22* gene is overexpressed in both strains leading to dysmyelination in the embryonic life followed by demyelination in about 26% of myelinated nerve fibers in adult mice^33^. These observations are in parallel with the finding in human CMT1A patient. Dysmyelination was detected in young patients with CMT1A without affecting the NCV whereas; demyelination was detected in adults CMT1A patients^34^. Therefore, we believe that these models are representative of CMT1A neuropathy and are good candidates for gene therapy studies^7^. Consistently, a 1.8-fold increase in Pmp22 protein levels was observed in the JP18 group and a threefold increase in the JP18/JY13 group when compared to wild-type B6 (WT) mice (Fig. 1a and Supplementary Fig. 6a). First, we examined CMT1A disease symptoms. A significant reduction in motor activity and weakness in muscular strength were observed in both strains compared with the WT mice (*p* < 0.001). The beam walking test showed a significant difference between the JP18 and JP18/JY13 strains (*p* < 0.05). However, this significance was not observed in the automated locotronic test, probably because the beam was harder to cross than the locotronic ladder (Fig. 1b and Supplementary Video 3). Concerning the electrophysiological endpoints, CMAP and sensory NCV were decreased in both transgenic strains when compared to the WT mice (*p* < 0.001) (Fig. 1c). Interestingly, the NCV values were comparable to patients with CMT1A pathology (<38 m/s)^35^. However, significant differences between both transgenic mouse strains were observed (*p* < 0.001 for CMAP and *p* < 0.05 for NCV), with JP18/JY13 mice being more affected by the neuropathy.

Results of the functional tests were supported by histological observations. Pathological hallmarks of CMT1A are alterations of the myelin sheaths and demyelination^35^. The number of myelinated fibers counted on semi-thin sections differed between all groups based on a quadratic regression analysis. The cutoff between small and large fibers was determined by fitting a quadratic statistical model for each group (Supplementary Table 2). Although statistical comparisons did not reach significance, a tendency towards a decrease in the number of the large fibers was observed in the JP18/JY13 mice (Fig. 1d). The ratio of the inner axonal diameter to the total outer diameter (g-ratio) is a widely used measure of axonal myelination. Histological analysis of ultrathin sections showed a significant increase in the g-ratios for both transgenic mouse strains when compared to the WT (*p* < 0.01 for J18 B6 and *p* < 0.001 for JP18/JY13 B6), reflecting myelin alterations consistent with a model of a demyelinating neuropathy (Fig. 1e). Notably, myelin sheaths were significantly narrower in JP18/JY13 mice when compared with JP18 mice (*p* < 0.001), highlighting the higher severity of the pathology in the JP18/JY13 strain. Moreover, the periodic spaces between thick dense lines were enlarged in both transgenic animal models (Fig. 1e) and paralleled the g-ratio increase. Overall, these data confirmed that the mouse models used showed disease markers remarkably comparable to CMT1A patients, which were dependent on the *PMP22* expression rate^32,36^. CMT1A patients exhibit severe, moderate or no signs of the disease depending on genetic and environmental factors^37^.

### siRNA PMP22-SQ NPs restore the functional and electrophysiological activity of JP18 and JP18/JY13 mice

Age-matched JP18 and JP18/JY13 mice were treated with five consecutives *i.v*. injections of siRNA PMP22-SQ NPs at the dose of 0.5 mg/kg twice per week (the treatment duration was 20 days, cumulative dose 2.5 mg/kg). The dose of the siPMP22-SQ NPs was calculated based on in vitro studies and previous studies done in our lab on prostate and thyroid carcinoma. The schedule of treatment was also chosen based on previous data from our research on cancer models^22,23,30^.

**Fig. 2:**
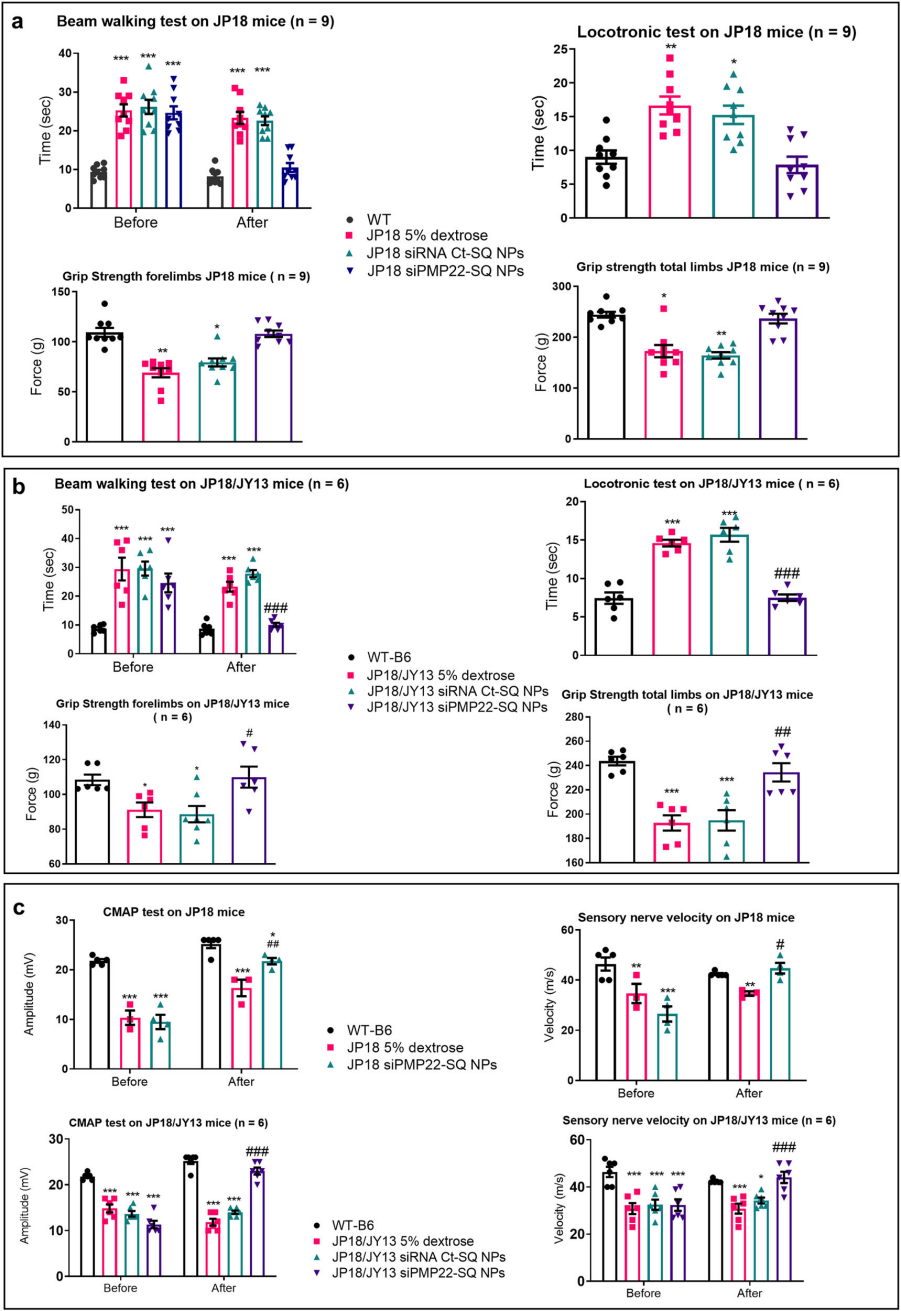
siRNA PMP22-SQ NPs treatment normalized the motor and sensory activity of both JP18 and JP18/JY13 CMT1A mice. Behavioral tests performed to study the motor activity of age-matched JP18 (**a**) and JP18/JY13 mice (**b**). CMT1A mice of both strains treated with siRNA PMP22-SQ NPs showed normalization of the time taken by the mice to walk across the beam or the locotronic ladder when compared to the WT and they were significantly faster and showed stronger grip strength than mice receiving 5% dextrose or siRNA Ct-SQ NPs. **c** Electrophysiological analysis showed that CMAP (left panel) and sensory NCV (right panel) were normalized for both JP18 and JP18/JY13 mice treated with siRNA PMP22-SQ NPs. Data represent mean ± s.e.m. Before: represent the analysis of the data before treatment. After: is the analysis of the test performed after the end of treatment. The JP18 and JP18/JY13 mice groups were divided blindly. Asterisk represents the significance between WT B6 and other groups. Hashtag represents significance between JP18 5% dextrose and the other two groups. *^,^^#^*p* < 0.05; **p < 0.01, ***^,^^###^*p* < 0.001 using ANOVA followed by Tukey’s multiple comparison tests.

Mice treated with siRNA PMP22-SQ NPs showed restoration and normalization of the motor activity and muscular strength (Fig. 2a, b and Supplementary Videos 3 and 4). Importantly, the siRNA Ct-SQ NPs had no effect on the same parameters. Moreover, treatment of JP18/JY13 mice displaying a very severe disease phenotype and paraplegia by siRNA PMP22-SQ NPs resulted in a significant improvement in motor activity (Supplementary Video 5). Noteworthy, upon treatment with siRNA PMP22-SQ NPs, the electrophysiological endpoints CMAP and sensory NCV were restored in both CMT1A mouse models and became even comparable to the WT group (Fig. 2c). Taken together, these data demonstrate that siRNA PMP22-SQ NPs induce remarkable recovery from CMT1A neuropathy, with the improvement of motor and electrophysiological parameters reaching levels observed in WT mice.

### siRNA PMP22-SQ NPs normalize Pmp22 expression and promote myelin and axon regeneration

**Fig. 3:**
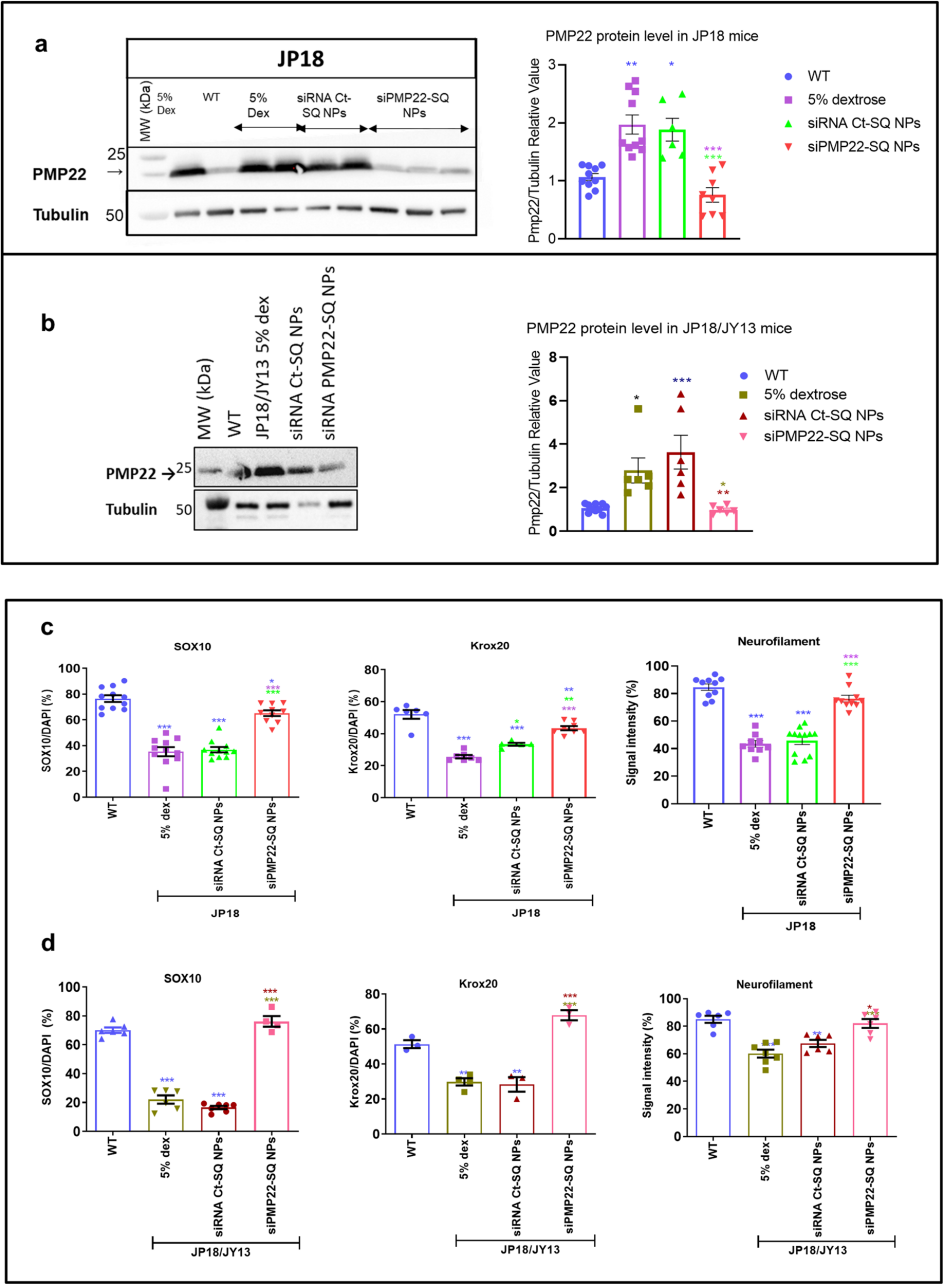
siRNA PMP22-SQ NPs inhibit Pmp22 protein expression and promote myelination and axonal regeneration factors. **a**, **b** Representative western blot showing the Pmp22 protein content of the different JP18 and JP18/JY13 treatment groups. Each well represents a different mouse. The protein quantification of Pmp22 was performed on at least five different mice per group for both strains. Pmp22 protein was normalized to tubulin as a reference protein. **c**, **d** Quantification analysis of immunohistochemistry images showing SOX10, Krox20, and Neurofilament on JP18 and JP18/JY13, respectively. SOX10 and Krox20 were normalized over DAPI expression to calculate their expression in percentage. Three different mice were analyzed per group. Data represent means ± s.e.m. Blue asterisks indicate significant differences between the WT and the other groups, purple asterisks indicate significant differences between 5% dextrose and the other groups, green asterisks indicate the differences between siRNA Ct-SQ NPs and the other groups and red asterisks the difference between siRNA-SQ NPs and the other groups. *p < 0.05; **p < 0.01, ***p < 0.001 using ANOVA analysis followed by Tukey’s multiple comparison tests.

We further investigated molecular markers of the functional recovery observed after siRNA PMP22-SQ NPs treatment. First, Western blot analysis showed that Pmp22 levels were normalized after treatment with siRNA PMP22-SQ NPs (respectively for JP18 and JP18/JY13, Fig. 3a, b, Supplementary Fig. 6b, c). Moreover, the protein levels of the major myelination proteins Myelin Protein Zero (P0) and Myelin Basic Protein (MBP) were not affected, indicating a specific inhibition of siRNA PMP22-SQ NPs without off-target gene effect (Supplementary Fig. 7a–f). Any change in P0 levels may lead to another demyelinating neuropathy, CMT1B^27^. This is in accordance with previous study showing that the myelin proteins were not affected by siRNA treatment in Tremble J mice^38^.

Since CMT1A is a demyelinating neuropathy, we also investigated the expression of two transcription factors, SOX10 and KROX20 (EGR2), both implicated in Schwann cell development and myelination^39^. Indeed, the *PMP22* gene harbors an intron site that is strongly activated by SOX10 and KROX20 and involved in the regulation of *PMP22* expression^40^. A significant decrease in the levels of the transcription factors was observed in both transgenic mouse models when compared to WT mice (*p* < 0.001 for SOX10 and KROX20 in JP18 mice; *p* < 0.001 for SOX10 in JP18/JY13 mice; *p* < 0.01 for KROX20 in JP18/JY13 mice). Upon treatment with siRNA PMP22-SQ NPs, levels of KROX20 and SOX10 statistically increased in the JP18 mice without reaching the WT level (*p* < 0.05), whereas in the JP18/JY13 mice their levels became comparable to WT mice (Fig. 3c, d and Supplementary Fig. 8a–c, respectively), supporting a direct involvement of both transcription factors in myelin recovery (Fig. 3c, d and Supplementary Fig. 8b, c, respectively, for JP18 and JP18/JY13).

A clinically highly relevant finding was that upon treatment with siRNA PMP22-SQ NPs, expression of the heavy 200-kDa neurofilament (NF-H), a marker of mature large diameter axons, was restored to WT levels (Fig. 3c, d and Supplementary Fig. 8a). A decrease in NF-H levels was observed in both transgenic mouse models (*p* < 0.001), and they increased upon siRNA nanoparticle treatment to levels observed in WT mice (right panels of Fig. 3c, d and Supplementary Fig. 8b, c, respectively, for JP18 and JP18/JY13). NF-H, in addition to providing structural support, plays a key role in axonal functions and nerve conduction^41^. Low levels of NF-H may contribute to axonal dysfunction and myelin abnormalities and are consistent with the decreased CMAP and sensory NCV values measured in JP18 and JP18/JY13 mice.

These data suggest that normalization of *PMP22* expression by siRNA PMP22-SQ NPs improves functional outcomes through both myelin and axon recovery. Furthermore, measures of CMAP and sensory NCV, reflecting neurophysiological recovery, may be considered as biomarkers of treatment efficacy. We thus histologically examined myelin and axon morphology in the sciatic nerves of siRNA-SQ NPs treated JP18 and JP18/JY13 mice.

The structure and density of the myelinated axons were first assessed on thionine blue-stained semi-thin sections from sciatic nerves. For either JP18 or JP18/JY13 mice, there were no significant differences in fiber counts between the siRNA PMP22-SQ NPs treatment group and the control groups (WT, dextrose or siRNA Ct-SQ NPs) (Supplementary Fig. 9a, b). However, for both JP18 and JP18/JY13 mice, elevated numbers of small myelinated fibers were counted in the mice treated with siRNA PMP22-SQ NPs. This may reflect decreased axonal diameter, axonal regeneration or compensatory axon sprouting as has been described for CMT^42,43^.

**Fig. 4:**
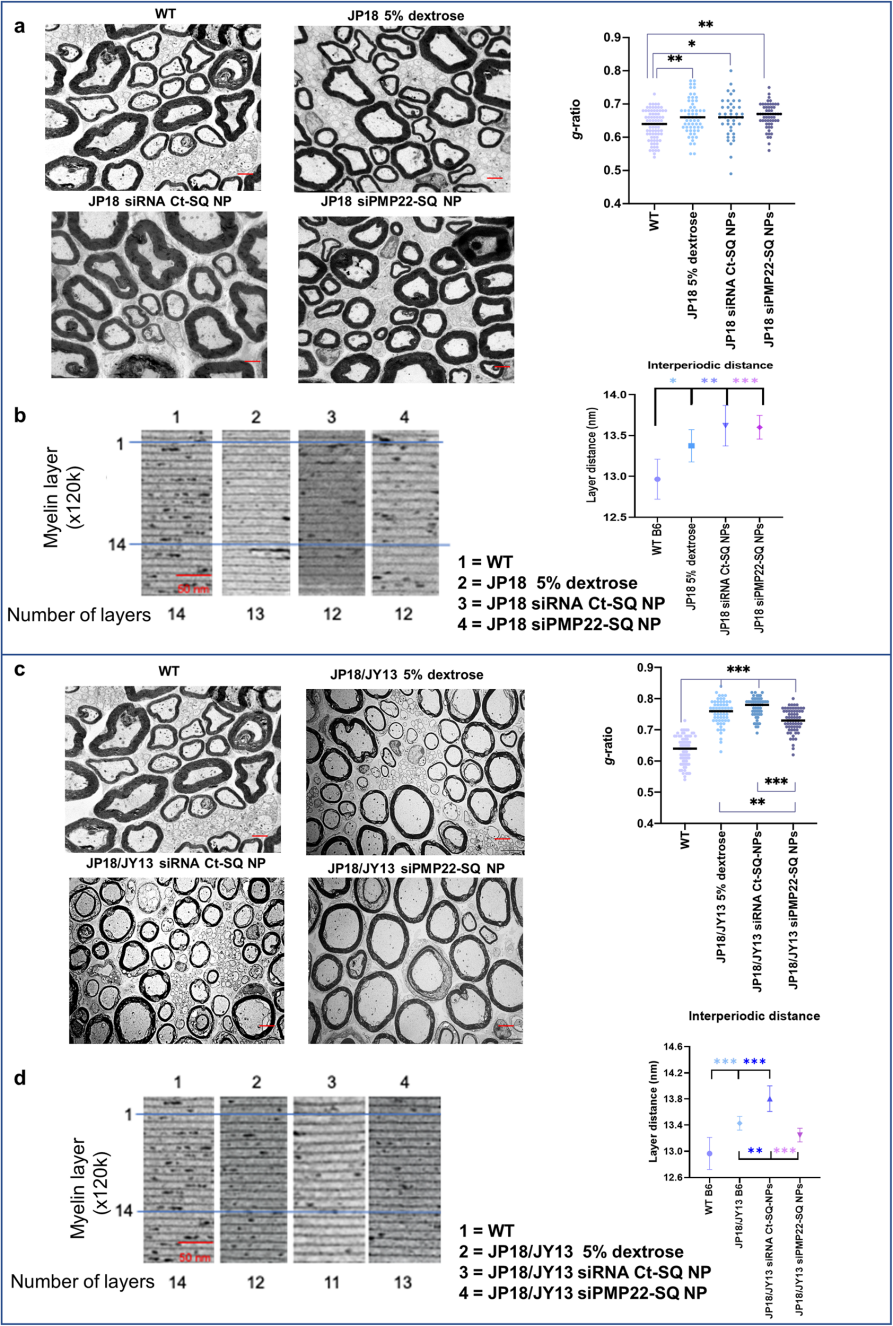
siRNA PMP22-SQ NPs are efficient to activate myelination. **a** Representative TEM images of ultrathin sections of sciatic nerves of WT (*n* = 5) and JP18 treated mice (*n* = 5) (×5000 magnification) followed by g-ratio analysis of myelinated fibers. Scale bar 2 µm. The bars represent the mean. **b** High magnification TEM images (×120 k) show myelin layer distance (Scale bar 50 nm) and its corresponding analysis. Data represent predicted means with 95% confidence intervals for layer distance analysis. The blue lines mark a zone of 14 myelin layers for the WT and show the difference between the JP18 groups. **c** Same analysis was performed for JP18/JY13 mice (*n* = 5 per group). siRNA PMP22-SQ NPs significantly decreased the g-ratio and **d** the inter myelin distance in JP18/JY13 mice. In JP18/JY13 mice, a compaction of the myelin layers distance was observed by the siRNA PMP22-SQ NPs and became identical to WT. The blue lines mark a zone of 14 myelin layers for the WT and show the difference between the JP18/JY13 groups. *p < 0.05; **p < 0.01; ***p < 0.001 using ANOVA analysis followed by Tukey’s multiple comparisons test.

Electron microscopic analysis of sciatic nerve sections provided further strong evidence for the therapeutic efficacy of siRNA PMP22-SQ NPs. In JP18 mice, nerve ultrastructure was less disturbed than in JP18/JY13 mice, with a higher *PMP22* copy number and a more severe disease phenotype (Fig. 4). There were no differences in g-ratios between the different JP18 treatment groups (Fig. 4a). The distances between individual myelin layers (interperiodic distances), representing the preservation of the myelin lamellar structure, followed the same pattern as the g-ratio results (Fig. 4b). The small changes in nerve fiber morphology did not reveal a structuring effect of siRNA PMP22-SQ NPs treatment in JP18 mice. On the contrary, alterations in myelin morphology were more marked in JP18/JY13 mice, with a significant increase in g-ratios, reflecting decreased myelin thickness, accompanied by a widening of the interperiodic distances (*p* < 0.001) (Fig. 4c, d). Knowing that the regulation of tight junctions and transmembrane adhesions are important functions of Pmp22, its abnormal expression is likely to result in alterations of myelin structure^44^. Treatment with siRNA PMP22-SQ NPs ameliorated both morphological parameters in the JP18/JY13 mice (i.e., g-ratio and interperiodic distances), however the g-ratio did not reach WT levels, most likely because of the short treatment duration (Fig. 4c). Interestingly, the interperiodic distance examination reached WT levels after siRNA PMP22-SQ NPs treatments (Fig. 4d).

This was translated by the improvement of nerve tissue architecture with large axons surrounded by regular and compact myelin testifying the functional recovery.

### siRNA-SQ NPs penetrate the sciatic nerve

**Fig. 5:**
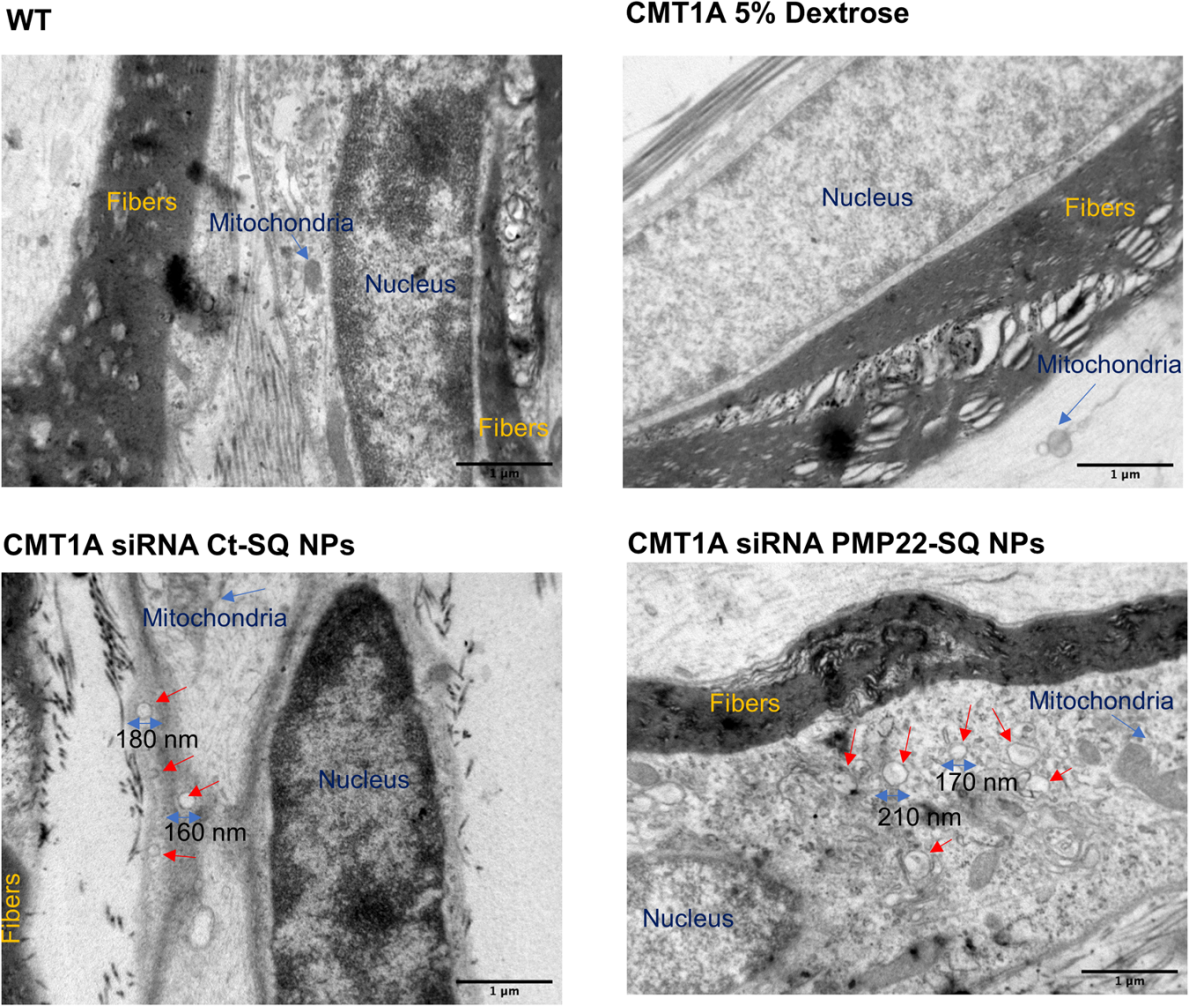
siRNA PMP22-SQ NPs and siRNA Ct-SQ NPs can reach the sciatic nerve and Schwann cells. Representative TEM images of ultrathin longitudinal sciatic nerves sections of ×25 k magnification of WT, CMT1A mice 5% dextrose, CMT1A siRNA Ct-SQ NPs and CMT1A siRNA PMP22-SQ NPs. The red arrows show vesicles having the same size as the nanoparticles in the CMT1A siRNA Ct-SQ NPs and siRNA PMP22-SQ NPs groups and, were found to be localized in the cytoplasm of Schwann cells. The blue arrows highlight the mitochondria that are differentiated from the vesicles. Scale bar 1 µm.

The examination of longitudinal nerve sections by electron microscopy revealed vesicles surrounded by a lipid layer with a size ranging from 160 to 210 nm, similar to the size of the siRNA-SQ NPs (Fig. 5). In contrast to transversal sections, it is possible to discriminate between these vesicles and neurotransmitter vesicles on longitudinal sections. Moreover, they could be easily differentiated from the mitochondria, in which the crescent-shaped interior could be seen (Fig. 5). Importantly, the small vesicles were only observed in nerve sections sampled from mice treated with siRNA SQ NPs. This allowed to hypothesize that after *i.v*. injection, the siRNA-SQ NPs interact with low-density lipoprotein (LDL) molecules present in the blood stream^45^ and transported inside the Schwann cells.

### siRNA PMP22-SQ NPs treatment results in a long-lasting effect over 3 weeks

**Fig. 6:**
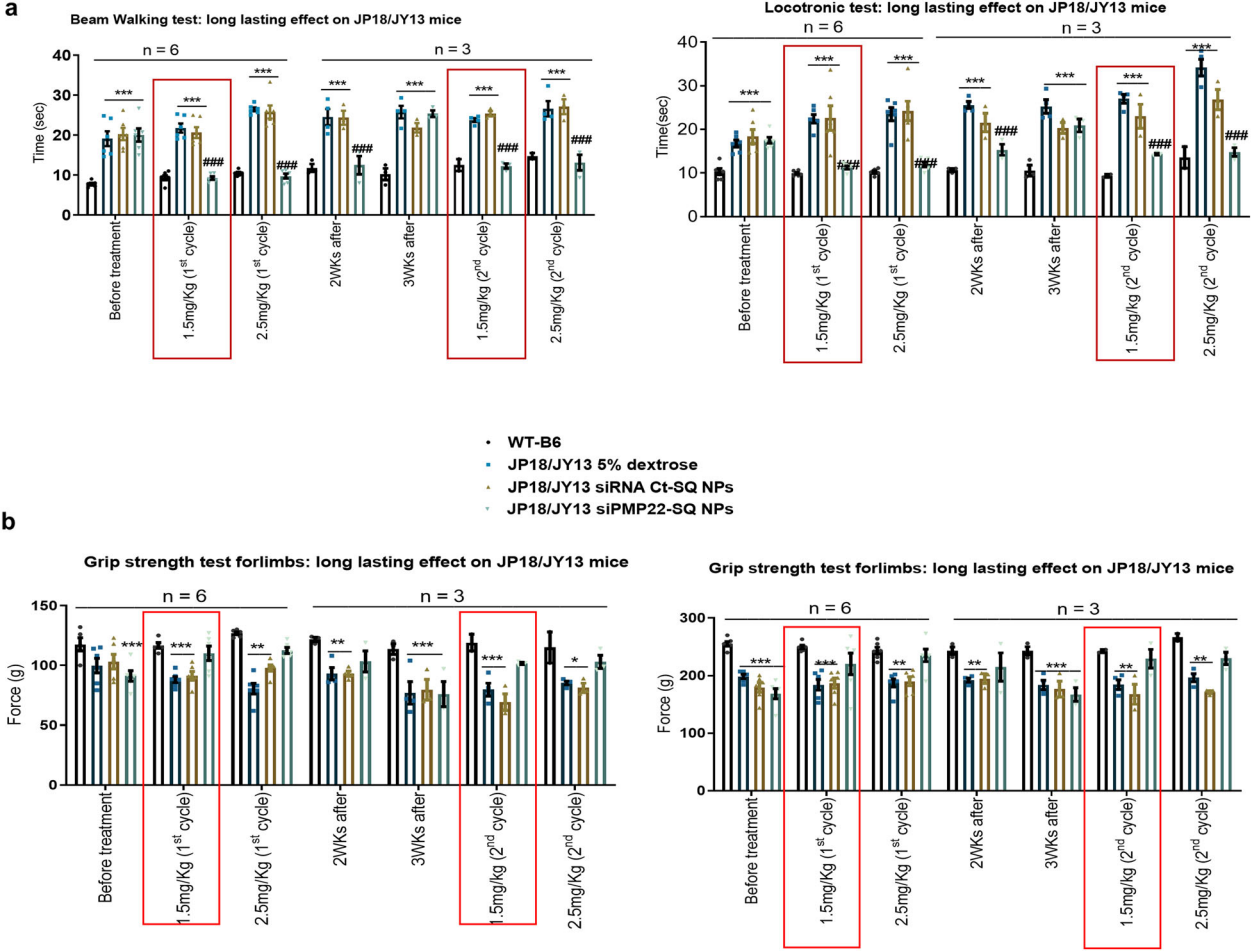
siRNA PMP22-SQ NPs have a long-lasting effect until around 3 weeks after the end of treatment. Behavioral tests analysis of long-lasting efficacy of siRNA PMP22-SQ NPs on JP18/JY13 mice. JP18/JY13 B6 mice received two cycles of treatment of 2.5 mg/kg siRNA PMP22-SQ NPs per cycle. Treatment was stopped for 21 days between the two cycles to investigate the relapse. The mice were followed weekly to analyze the recovery and relapse periods. **a** Represents the time in seconds taken by the mice to perform the beam walking test and locotronic test. **b** Represents grip strength force analysis on the forelimbs and the total limbs. The red box highlights the efficient dose of 1.5 mg/kg at which the mice were able to perform better on both the motor and muscular activities. Before treatment: corresponds to the data analyzed before starting the treatment of each group that were chosen blindly. After 2 and 3 weeks: correspond to the data collected after stopping treatment during 2 and 3 weeks. Data represents mean ± s.e.m. Asterisk represents the significance between WT B6 and other groups; hashtag represents the significance between JP18/JY13, 5% dextrose and JP18/JY13 siRNA PMP22-SQ NPs. **p < 0.01, ***p < 0.001 (ANOVA analysis followed by Tukey’s multiple comparisons test).

After demonstrating that dosed siRNA PMP22-SQ NPs treatment restored peripheral nerve functions and structure and especially promoted locomotor recovery and muscle strength with a remarkable efficacy in the two mouse models of moderate and severe CMT1A, we investigated the long-lasting efficacy of this precision therapy. As in the previous experiments, JP18/JY13 mice aged 12 weeks were treated with either 5% dextrose, siRNA Ct-SQ NPs or siRNA PMP22-SQ NPs (2.5 mg/kg cumulative dose divided into five injections at 3 days interval) and compared to WTs. They were followed until the relapse period and then reinjected till functional recovery (Supplementary Fig. 10, Step 5). When the cumulative dose of siRNA PMP22-SQ NPs reached 1.5 mg/kg (after three injections), locomotor activity and muscle strength were already restored (Fig. 6a, b). After the last injection (5th injection), the positive effects of the siRNA PMP22-SQ NPs lasted for a period of 3 weeks. A second cycle of treatment was then started, and again full locomotor recovery and muscle strength were reached at the cumulative dose of 1.5 mg/kg (Fig. 6a, b). The two treatment cycles showed similar efficacy and did not affect body or organ weight as well as liver enzymes (AST and ALT) and kidney function (plasma creatinine and albumin) (Supplementary Table 3). The toxic effects of siRNA PMP22-SQ NPs were investigated in these two main excretory organs that showed high accumulation of siRNA-SQ NPs in our previous study^30^. In addition, since SQ is a precursor of cholesterol^46^, the levels of different forms of plasma cholesterol were studied and we found no significant change in their levels between the treated groups (Supplementary Table 3). Observation on TEM images showed no toxicity of the siRNA-SQ NPs on the sciatic nerve represented by the absence of Schwann cell damage, no alterations in the number of mitochondria and no modifications in the intracellular organelles.

## Discussion

The recent approval of the first-ever siRNA-based drug of the treatment of a neurological disease, hereditary transthyretin-mediated amyloidosis, has paved the way for a new therapeutic approach based on post-transcriptional disease gene silencing^47,48^. The therapeutic siRNA exerts its beneficial effects by inhibiting hepatic transthyretin production. The liver is indeed the main source of circulating transthyretin, and accumulation of the mutant protein in peripheral nerves forms amyloid deposits leading to rapidly progressing polyneuropathy.

The present study broadens the therapeutic use for siRNA for diseases of the nervous system. We used a hydrophobic siRNA-SQ bioconjugate forming NPs that allowed us to target a diseased gene within the nervous system. Importantly, we provide proof of concept that the dosed administration of nanoparticle-stabilized siRNA allows to normalize the expression of a dosage-sensitive gene. We show that the dosed administration of siRNA PMP22-SQ NPs normalizes the expression of Schwann cell-specific PMP22 in two preclinical CMT1A mouse models, without an off-target effect on P0 or MBP, re-establishes electrophysiological activity of both motor and sensory nerves and results in the rapid recovery of locomotor functions and muscular strength of the limbs. Synthetic ASO against PMP22 have also been developed recently with encouraging results in CMT1A animal models^14^. However, barriers to their use are off-target actions and toxic side-effects during long-term treatment^49^. The siRNA-SQ NPs seem to have less off-target actions after long-term treatment due to the absence of phenotypic organ toxicity and unaffected biochemical markers. Nevertheless, a deeper toxicological study should be performed.

The solely study using siRNA specific for PMP22 was initiated in the Trembler-J mouse mutant^38^ where mutation of Leu^16^Pro is responsible for the development of CMT1E. In this study, the intraperitoneal injection to postnatal day 6 Tr-J mice resulted in positive outcomes. However, the authors did not investigate the treatment effect in adult mice by siRNA injection via *i.v*. route instead of intraperitoneal injection. In addition, our data are consistent with two recent studies showing that inhibiting PMP22 expression can ameliorate the neuropathological symptoms caused by *PMP22* overexpression^50,51^. In contrast with our study, where siRNA was injected via *i.v.* route; both studies used as oligonucleotide delivery intraneural injections, a route hard to be applied in human treatment.

In our study, the functional recovery was accompanied by the increased expression of neuronal (NF-H) and glial (Sox10, KROX20) markers and by the structural improvement of nerve fibers with the concurrent normalization of g-ratios. These results were remarkable as PMP22 expression needs to be fine-tuned, its overexpression resulting in CMT1A, when too low levels lead to HNPP. Noteworthy, there was no immediate decline in the pharmacological efficacy of siRNA PMP22-SQ NPs as effects lasted for at least 3 weeks after ending the treatment. Moreover, after interruption, the treatment could be initiated again with success.

Therefore, treatment with siRNA PMP22-SQ NPs represents a potent promising therapy for CMT1A patients. The normalization of gene expression by specific siRNA conjugated to NPs also offers new perspectives for the treatment of the wide range of diseases, in particular nervous system disorders, caused by unbalanced genomic arrangements resulting in copy-number variations^52,53^. Possible applications of this therapeutic approach could go beyond the treatment of genetic diseases and may be extended to the normalization of gene expression altered by environmental factors, lifestyles, and age-related disorders.

## Methods

### Screening of siRNA against PMP22

To restore the basal levels of *PMP22* gene expression, we first obtained the mRNA PMP22 sequence for both *homo sapiens* and *mus musculus*, then defined the homology part between these two sequences (Supplementary Table 1a). Different siRNAs were designed and blasted to check their specificity. The siRNAs were checked for scoring by using three different methods (Tafer software, Thermofisher software and Reynold method). Reynold scores were calculated respecting different criteria^25^. Eight siRNAs against PMP22 (Supplementary Table 1b) were chosen to continue the study, in addition to a siRNA control (siRNA Ct, a scrambled sequence).

### siRNAs and chemical modifications

The designed sequences of sense and antisense siRNA strands were purchased from Eurogentec, France. siRNAs were characterized by matrix-assisted laser desorption/ionization time-of-flight mass spectrometry (MALDI-TOF MS) then purified by Reverse Phase-High-Performance Liquid Chromatography (RP-HPLC). Single-stranded RNAs were synthesized as 19-mers with two 3′-overhanging 2′-deoxynucleotides residues to increase their stability as described by Tuschl et al.^54^. To allow conjugation with SQ, a DBCO reactive group was introduced at the 5′-end of the sense strand of each siRNA sequence through a *N-*(hexamethylenyl)-6-oxohexanamide spacer (C_6_). To generate siRNA from RNA single strands, equimolar amounts of both sense and antisense strands were annealed in annealing buffer [30 mM HEPES-KOH (pH 7.4), 2 mM Mg acetate, 100 mM K acetate] for 3 min at 95 °C and then incubated for 45 min at room temperature before storing at −80 °C.

### Bioconjugation of siRNA

To avoid any degradation of the siRNA by ribonucleases, precautions were taken before each synthesis. The bioconjugates siRNA-SQ were obtained by the copper-free 1,3–dipolar cycloaddition of azido-SQ with DBCO derived siRNAs sense strand^30^. The protocol to obtain the bioconjugates siRNA-SQ was slightly modified and described as follows. One nmol of the 5′-end modified sense strand DBCO-C_6_ of the siRNA (1 mg/mL in DNAse/RNAse-free water) was mixed with 50 nmol of SQ-N_3_ (1 mg/mL in DMSO) in a glass vial containing DMSO (286 µL) and acetone (65 µL). The solution was then incubated at room temperature for 12 h under stirring to obtain the bioconjugate sense-strand siRNA-SQ. In the following day, excess acetone was eliminated under nitrogen flow for 30 min, followed by lyophilization for 24 h to remove the solvents. Purification of the bioconjugate from the excess of unconjugated SQ was performed by RP-HPLC on a polymeric column. Purified products were lyophilized, and then solubilized in RNAse-free water at the desired molar concentration.

### Purification of siRNA-SQ bioconjugates via HPLC

HPLC purification was performed on a Thermo scientific high-performance liquid chromatography system (Dionex UHPLC-3000) equipped with a photodiode array detector (DAD-3000) whose wavelength ranged between 190 and 800 nm, a pump and a manual injector. The stationary phase consisted of a nonporous, alkylated polystyrene divinylbenzene column (Hamilton PRP-3 10 μm, 4.6 × 250 mm, PEEK, Ref: 79574) protected by a pre-column (Hamilton). Thermofisher Chromeleon 7 software was used for data acquisition. Flow rate was of 1.2 mL/min and injection volumes of 100 μL. A gradient of mobile phases A and B was applied. Mobile phase A was composed of 0.2 M TEAA (5%), pH 7.0, with 5% acetonitrile, water 90%, while mobile phase B consisted of 90% acetonitrile with 5% TEAA, 5% of water. The gradient applied was as follows: 0–8 min linear gradient from 0 to 24% of phase B; 8–16 min linear gradient from 24 to 90% of phase B; 16–18 min linear gradient from 90 to 100% of phase B; 18–30 min 100% of phase B; 30–32 min linear gradient from 100% of phase B to 100% of phase A and 32–42 min re-equilibration with 100% phase A. Bioconjugates sense-strand siRNA-SQ were purified by manual peak collection. Fractions were collected for 2 min, corresponding to a fraction volume of 2.4 mL, and then lyophilized. All lyophilized siRNA fractions were reconstituted in RNAse-free water.

### MALDI-TOF mass spectrometry

A MALDI-TOF/TOF UltrafleXtreme mass spectrometer (Bruker Daltonics, Bremen) was used for all experiments to verify the identity of the obtained bioconjugates. Mass spectra were obtained in linear positive ion mode. The laser intensity was fixed just above the ion generation threshold to obtain peaks with the highest possible signal-to-noise (S/N) ratio without a significant broadening of the peaks. All data were analyzed using the Flex Analysis software package (Bruker Daltonics).

### Annealing of siRNA-SQ bioconjugates to antisense siRNA strands

Annealing of both strands of siRNA PMP22 and siRNA Ct was performed after the bioconjugation of the sense strand to SQ and is the same as mentioned before for the generation of siRNA from single RNA strands. Precisely, equimolar amounts of both sense-strand siRNA PMP22-SQ bioconjugate and antisense siRNA PMP22 were mixed with an annealing buffer [30 mM HEPES-KOH (pH 7.4), 2 mM Mg acetate, 100 mM K acetate] and incubated at 95 °C for 3 min, then incubated for 45 min at room temperature. The same protocol was performed to obtain annealed siRNA Ct-SQ bioconjugate which is represented in Supplementary Fig. 10, step 1.

### Preparation and characterization of nanoparticles siRNA-SQ

NPs siRNA PMP22-SQ and siRNA Ct-SQ were prepared by nanoprecipitation in acetone: water (volume ratio 1:2). One phase was slowly added to the other, under stirring, i.e., 10 nmole of siRNA-SQ was dissolved in 1 mL of RNase-free water and added drop wisely over 500 µL of acetone under stirring. Then the solution was incubated under stirring for 5 min after which acetone was completely evaporated using nitrogen flux to obtain an aqueous suspension of pure siRNA-SQ NPs at desired concentration.

The hydrodynamic diameter (nm) of the obtained siRNA-NPs was measured by dynamic light scattering Malven Zeta Sizer NANO. Samples were analyzed at 10 µM concentration in H_2_O. Three measures of 5 min for each sample were performed and the average diameter ±S.D. of at least three independent samples was calculated.

Cryogenic Transmission Electron Microscopy (cryo-TEM) was performed with the JEOL 2100 electron microscope at the Electronic Microscopy Platform (IBPS/Institut de Biologie Paris-Seine, Université P. et M. Curie, Paris, FRANCE). 4 μL of siRNA PMP22-SQ NPs (concentration of 2.2 mg/mL) was placed on a carbon-coated copper grid. A filter paper was used to remove the excess solvents, and the samples were directly dipped in liquid ethane solution using a guillotine-like frame and transferred to a cryo-sample holder. The siRNA-SQ NPs were observed at an acceleration voltage of 200 kV under a low electron dose. Analysis was performed with Image J software.

### Cell line

MSC80 cell line (mouse Schwann cell line) that expresses myelin genes *PMP22* and *P0* was used in this study^55^. Cells were grown in a DMEM medium (1X) (Gibco Life Technologies Ref: 11960-044) supplemented with 10% fetal bovine serum, (Gibco Life Technologies Ref: 10500-064), 1%Penicillin/Streptomycin (Gibco Life Technologies Ref: 15070-063), 1%Sodium Pyruvate (Thermo Scientific Ref: SH30239.01), 1% l-glutamine 100X, (Life Technologies Ref: 25030-024) and 2.5 µg/ml of Fungizone Amphotericin B, (Gibco Life Technologies Ref: 15290-026). Cells were incubated at 37 °C in a humidified atmosphere containing 5% CO_2_.

### In vitro cell transfection

To choose the most efficient siRNA PMP22, 3 × 10^5^ MSC80 cells were seeded in six-well plates containing complete medium until 60–70% confluency. Then, transfection was carried out using Lipofectamine 2000^®^ according to the manufacturer’s instructions in Opti-MEM reduced serum-free medium. Eight different siRNAs PMP22 and siRNA Ct were transfected at 50 nM concentration. Four hours later, the medium was replaced with a complete DMEM medium. After 48 and 72 h, cells were harvested, then mRNA and proteins were extracted to determine gene and protein expression.

After choosing the most efficient siRNA PMP22 sequence based on the different criteria, MSC80 cells were seeded and transfected with different concentrations of siRNA PMP22 (25, 50, and 100 nM). Cells were collected after 48 and 72 h for gene expression analysis. To study the efficacy of siRNA PMP22-SQ NPs, the same protocol as mentioned above was performed. Each experiment was performed at least three times in duplicates.

### mRNA extraction and real-time PCR (RT-qPCR)

Total RNA was extracted from MSC80 cells using RNeasy mini-kit (Qiagen, Courtaboeuf, France). First-strand cDNA was generated with M-MLV RT buffer pack (Invitrogen, Charbonnières-les-Bains, France). Real-time PCR (qPCR) was carried out using StepOnePlus PCR System (AB Applied Biosystems, Villebon-sur-Yvette, France) with Maxima Syber Green Rox qPCR master Mix (Thermo Scientific, Villebon-sur-Yvette, France), according to the manufacturer’s instructions. Each experiment was performed at least three times in triplicate. Gene expression was determined by 2^−ΔΔCt^ method and normalized to 18S levels. Relative mRNA expressions of targeted genes were compared to non-treated cells.

### Western blot analysis

Total protein extracts from both MSC80 cells and sciatic nerve tissue were obtained using RIPA buffer (Sigma R0278) supplemented with a protease inhibitor cocktail (Roche, Neuilly sur Seine, France). Sciatic nerve tissues were first homogenized using Precellys 24^®^ lysis and homogenizer (Bertin, Technologies, France). All samples (cells and tissues) were then incubated for 2 h, under rotation at 4 °C for complete protein extraction and then centrifuged at 13,000 rpm for 20 min. Supernatant was stored at −80 °C.

Proteins were quantified by BioRAD Assay according to the manufacturer’s instructions. Practically, 20 µg of MSC80 protein and 5 µg of sciatic nerve proteins were loaded on a gradient polyacrylamide gel of 8–12% (Invitrogen, Ref: NW04120) and then transferred to a nitrocellulose membrane using the iBlot Dry Blotting System (Invitrogen iBLOT 2, Cat# IB21001). Membranes were blocked with 5% BSA solution for 1 h and then incubated overnight at 4 °C with the following primary antibodies: anti PMP22 (1:750, Sigma: SAB4502217), anti-myelin protein zero (P0) (1/1000 abcam: ab31851), anti-MBP (1:1000, Abcam: ab7349), monoclonal GAPDH-HRP linked (1:1000, Cell Signaling technology, Saint Quentin in Yvelines, France. Ref: 3683), and monoclonal anti tubulin (1:10000, Cell signaling: mAb#3873). Blots were then washed and incubated with the corresponding secondary anti-rabbit and anti-mouse antibody conjugated to HRP (horseradish peroxidase, 1:3000, Cell Signaling technology) for PMP22, P0, MBP, and tubulin, respectively. Bands were visualized by enhanced chemiluminescence reagent (BioRAD, France) and quantified using image J software.

### Cell viability test

The efficacy of siRNA PMP22, siRNA Ct, siRNA PMP22-SQ NPs, and siRNA Ct-SQ NPs were tested for viability on MSC80 cells using MTT assay. A total of 20 × 10^3^ MSC80 cells were seeded in 96-well plate containing complete medium. When the cells reached 60–70% confluency, they were transfected using lipofectamine 2000 as mentioned before. After 72 h, the cells were incubated for 2 h at 37 °C with MTT solution according to the manufacturer’s instructions. Then, the medium was removed and replaced by DMSO. Absorbance was read at 570 nm and the mean of three independent experiments was recorded ±standard error of the mean (s.e.m.).

### CMT1A mice models

Two separate lines of transgenic mice JP18 and JY13 established by Perea et al.^32^ were used in this study. Transgenic mice were purchased after reviviscence from “Transgenesis, Archiving and Animal Models (TAAM-UPS44)”. Both strains were archived by cryopreservation and generated on the C57BL/6J (B6) background.

For the JP18 mouse model, the mouse *PMP22* cDNA was introduced under control of the PhCMV*-1 promoter, therefore, mice overexpressed *PMP22* throughout life. The JY13 mouse model carried the intact human *PMP22* gene under a tTA open reading frame that gave Schwann cell-specific expression of *tTA*^32^. JY13 strain was found to have little adverse effect on myelination. Double transgenic mouse model (JP18/JY13) was generated by crossing JP18 and JY13 mice. In absence of tetracycline, *PMP22* overexpression occurred throughout the mice lifespan^32^.

The mice were genotyped at day 7 after birth by using specific primers of *PMP22* and *tTA* genes already described by Perea et al.^32^. After PCR, the samples were run on 1.5% agarose gel and the bands were visualized under UV light.

### Experimental approach

The experimental approach and the performed biological and chemical studies are presented in Supplementary Fig. 10, steps 2 and 3. According to Perea et al., we used JP18 mice at 16 weeks of age. This age showed a sign of pathology with demyelinating fibers^32^. The animals were randomly assigned to each group before performing the behavioral and the electrophysiological tests. Therefore, age-matched JP18 B6 mice were divided into three groups of nine mice each. Group one received a vehicle of 5% dextrose solution, mice of group two were treated with siRNA Ct-SQ NPs and mice of the third group were treated with siRNA PMP22-SQ NPs. In addition, one group of WT B6 mice were used as a control (nine mice). All the treatments were administered by retro orbital *i.v*. injection, following a regular treatment schedule, i.e., a cumulative dose of 2.5 mg/kg at an interval of 0.5 mg/kg per injection twice per week. Beam walking test was performed before and after treatment while locotronic and grip strength tests were performed after treatment. After treatment, the body weight, heart and kidney were weighed. Sciatic nerves were collected for further studies. Blood was collected directly from the heart for biochemical analysis and analyzed in the Department of Biochemistry by Pr. Patrice THEROND. The experiment was repeated twice and the results were combined.

In another similar experiment, to test siRNA PMP22-SQ NPs on a more affected CMT1A mouse model, the double transgenic model (JP18/JY13) was used that harbored two extra copies of *PMP22* gene. At 12 weeks of age, JP18/JY13 mice were divided into three groups, similar to the groups of JP18 mice. The same protocol of treatment was performed and 6 mice per group were used, in addition to a WT B6 group (*n* = 5). Behavioral tests were performed as mentioned for the JP18 B6 mice group and sciatic nerves were collected afterward. Biochemical analysis were performed as for JP18.

To study the long-lasting effect of siRNA PMP22-SQ NPs, JP18/JY13 B6 mice of 12 weeks age were used. Mice were again divided into three groups of six mice each: JP18/JY13 vehicle, JP18/JY13 siRNA Ct-SQ NPs, and JP18/JY13 siRNA PMP22-SQ NPs in addition to a WT B6 group as a control (*n* = 5). Two cycles of treatment were administered (Supplementary Fig. 10, step 5). The first cycle was using a cumulative dose of 2.5 mg/kg of siRNA PMP22-SQ NPs and siRNA Ct-SQ NPs at an interval of 0.5 mg/Kg per injection, twice per week. Then, treatments were stopped for 3 weeks to check the relapse period. After relapse, a new cycle of treatment was initiated for another cumulative dose of 2.5 mg/kg of siRNA-SQ NPs at an interval of 0.5 mg/Kg per injection twice per week. Behavioral tests were performed before treatment, at 1.5 mg/kg of first treatment cycle, at 2.5 mg/kg of first treatment cycle, 2 weeks after stopping the first cycle treatment, 3 weeks after stopping the first cycle treatment, at 1.5 mg/kg of second treatment cycle and at 2.5 mg/kg of second treatment cycle.

The number of animals per group, are in accordance with the 3R rule that aims to reduce the use of animals in preclinical research. All animal experiments were approved by the institutional Ethics Committee of Animal Experimentation and research council, registered in the French Ministry of Higher Education and Research « Ministère de l’Enseignement Supérieur et de la Recherche; MESR, autorisation N°: APAFIS#10131-2016112916404689 ». It is carried out according to French laws and regulations under the conditions established by the European Community (Directive 2010/63/UE). Investigation has been conducted in accordance with the ethical standards and according to the Declaration of Helsinki. All efforts were made to minimize animal suffering. Administration of treatments was performed under isoflurane anesthesia and animals were sacrificed by cervical dislocation. All animals were housed in sterilized laminar flow caging system. Food, water, and bedding were sterilized before animals were placed in the cages. Food and water were given ad libitum.

### Beam walking test

To test the gait of JP18 and JP18/JY13, the mouse beam walking test was performed (Supplementary Fig. 10, step 3). Mice were allowed to walk on a platform with a rod of 3 cm diameter, 70 cm length and around 30 cm above a flat surface^56^. At one end of the rod, a secure platform was set to house the animal. First, the mouse was allowed to adapt and then trained to cross the beam from one side to the other. The time to cross the platform was recorded for analysis. Each animal was tested for three trials per session, before starting the treatment and after the treatments protocols as detailed above. For the long-lasting efficacy experiment, beam walking test was performed at different time points. The test was repeated three times per animal and was recorded by a camera. Data were presented as average time spent by the mice per group ± s.e.m.

### Locotronic test

The locotronic apparatus (Intellibio innovations A-1805-00049) was used to test the motor coordination when walking (Supplementary Fig. 10, step 3). The mice were allowed to cross a 75 × 5 × 20 cm horizontal ladder with bars (7  mm in diameter), which were set 2 cm apart. Infrared photocell sensors situated above and below the bars monitored paw errors. The locotronic apparatus was linked to a software that automatically recorded the time taken by the mouse to cross the path. The time was assessed in three trials, with 20 min rest between trials. The statistical analysis of the data was performed by calculating the mean of three trials for each animal. Data are presented as the mean ± s.e.m. For the regular treatment, the test was performed at the end of the treatment, while for the long-lasting experiment it was performed at different time points.

### Grip strength test

Neuromuscular strength was assessed by using the Grip strength test (BIOSEB Innovation Model: BIO-GS3) (Supplementary Fig. 10, step 3). This test was performed using an automated grip strength meter. The apparatus consisted of a T-shaped metal bar and a rectangular metal bar connected to a strength transducer. To measure strength in the forepaws of the mice, each mouse was held gently by the base of the tail, allowing the animal to grasp the T-shaped metal bar with its forepaws. As soon as the mouse grasped the transducer metal bar with forepaws, the animal was pulled backwards by the tail until grip was lost. This step was repeated three times and the highest strength was automatically recorded in grams (g). To measure the strength on both limbs, each mouse was allowed to grasp the rectangular metal by the fore and hind limbs. After that, it was gently pulled by its tail perpendicular to the axes of the apparatus until the animal lost the grip. The highest strength was recorded automatically. For the regular treatment protocol, the test was performed at the end of the treatment, while for the long-lasting experiment it was performed at different time points as detailed above. Data represent average strength per group ± s.e.m.

### Electrophysiological test

The test was performed with a standard EMG apparatus (Natus UltraPro S100 EMG) in accordance with the guidelines of the American Association of Neuromuscular and Electrodiagnostic Medicine. Anesthesia was performed by isoflurane inhalation where mice were placed in an induction chamber containing 1.5–2% isoflurane in pure oxygen. During the whole procedure, anesthesia was maintained on the same level through a face mask. Mice were placed on their frontal side on a heating pad to maintain their body temperature between 34 and 36 °C. For recording the CMAP, three needles were inserted in mice thigh: the stimulator needle electrode at the sciatic nerve notch level, the anode electrode in the upper base part of the tail, while the receptor needle (or recording needle) was inserted in the medial part of the gastrocnemius muscle. A supramaximal square wave pulse of 8 mA was delivered through the stimulator needle and recorded through the muscle as amplitude. For the measurement of sensory NCV, multiple stimulation of the caudal nerve was delivered through the stimulator needle that was located at the 2/3 of the length of the tail, at a distance of 2–2.5 cm from the receptor needle. The ground electrode was inserted half-way between the stimulator and the receptor electrodes. The sensory NCV was calculated from the latency of the stimulus and the distance between the stimulator and receptor electrodes^57^.

### Sacrifice and organ collection

Body weight of each mice was measured before sacrifice and the heart and kidney were weighed to measure organ hypertrophy. Sciatic nerves were taken and used for the histological and protein investigations (Supplementary Fig. 10, step 4).

### Investigation of myelination and axonal regeneration markers by confocal microscopy

Sciatic nerves were incubated in 4% paraformaldehyde solution at 4 °C for 2 h for fixation. After that, samples were washed three times 5 min each with phosphate buffer solution, followed by 5 min incubation with 5% sucrose prepared in PBS. Then, overnight incubation with 20% sucrose was done. In the next day, each sciatic nerve was embedded in O.C.T. Tissue-Tek (Sakura 4583) and immediately snap-frozen in ice cold isopentane solution, placed in liquid nitrogen and then stored at −80 °C. Frozen sections of 5 µm thickness were prepared with a cryostat at −20 °C and placed on (3-Aminopropyl) triethoxysilane (Merck, 440140) coated super frost slides. Slides were either used directly or stored at −80 °C.

Before starting the permeabilization step, the slides were allowed equilibrating at room temperature for ~30 min. For nuclear proteins SOX10 and KROX20, a permeabilization step was performed using 0.2% Triton X and 0.1% Tween 20 solution in PBS 1X for 30 min. Samples for cytoplasmic protein NFs were permeabilized using ice cold methanol for 10 min. All samples were then blocked for 1 h using blocking buffer consisting of 2% BSA, 0.1% Tween 20 and 5% FBS (replaced by normal donkey serum for SOX10). Subsequently, samples were immunostained for markers specific for myelinating Schwann cells (Egr2 or Krox20), myelinating and non-myelinating Schwann cells (Sox10) and axonal fibers (NF). Primary antibodies listed in Supplementary Table 4 were prepared in their respective blocking buffer and samples were incubated overnight at 4 °C. Then, samples were washed by PBS 1X three times 5 min each, followed by complementary secondary antibodies incubation (Supplementary Table 4) for 1 h at room temperature. Afterwards, three washes of PBS 1X 5 min each were done. Cell nuclei were stained with 5 µg/ml DAPI in PVA mounting medium (Inova Diagnostics, San Diego, CA). Digital images were obtained with an Olympus IX70 fluorescence microscope (Olympus, Tokyo, Japan) equipped with Leica DFC340 FX camera using Leica Application. Images were analyzed using image J software.

### Exploration of fibers count and g-ratio

Sciatic nerves were incubated in 3.6% glutaraldehyde solution (Sigma-Aldrich cat# G5882) for 4 h at 4 °C. This was followed by a PBS 1X wash for 5 min and a post fixation step in 2% osmium tetroxide (Sigma-Aldrich cat# 251755-5 ml) for 2 h. Dehydration step consisted in 5 min washes, using respectively 50%, 80%, 95% and 100% ethanol. The samples were further incubated in acetone solution for 15 min twice, then in an acetone-EPON solution (50:50) for 15 min, followed by embedding in EPON solution twice for 30 and 60 min, respectively. Finally, the samples were carefully positioned in molds filled with liquid epoxy resin solution consisting of 25 mL EPON (Fluka, cat# 45345), 11 mL DDSA (Fluka, cat#45346), 15 ml MNA (Fluka, cat#45347), 0.70 mL DMP30 (Fluka, cat# 45348) in the desired orientation, either for transverse or longitudinal sections and polymerized for 48 h at 60 °C.

For thionine blue staining, semi-thin sections of 1 µm thickness of sciatic nerves were prepared using ultra microtome (Ultratome, Leica, Germany) and sections were stained with thionine blue for 1 h at 60 °C followed by washes of 100% ethanol and xylene, respectively. Slides were covered with a cover slip using a mounting medium (Eukitt, Merck, France). Tissues were finally scanned by optical Microscopy and analyzed for fiber diameter density.

For transmission electron microscopy, ultrathin sections of 70 nm were prepared using ultra microtome (Ultratome, Leica, Germany) and contrasted with uranyl acetate solution and lead citrate. Sections were analyzed using a 1010 electron microscopy (JEOL, Japan) and a digital camera (Gatan, US). Images were used to calculate the g-ratio and the interperiodic distance of myelin sheath.

### Statistics and reproducibility

Statistics were computed with GraphPad Prism 8.3.0 software. Outliers were identified using Grubbs’ test. Differences in group means were calculated by one-way ANOVA followed by Dunn’s multiple comparison test. For studies requiring grouped analyses, a one-way ANOVA followed by Tukey’s multiple comparison test was performed. When we have two groups to compare, a Mann–Whitney analysis was performed to assess the statistical difference. A value of *p* < 0.05 was considered significant. All measures were taken from distinct samples, and the sample sizes are presented in figure legends. All the in vitro experiments were performed at least three times independently. Data are presented as mean ± s.e.m.

To define the cut-offs of fibers diameter the relationship between fiber counts (after square root transformation) with diameter (after log transformation) was modeled using a quadratic model where predictions from this model were used to identify the cutoff. The estimated quadratic equations, where parameters defining the equation wee estimated using least squares regression, are presented in Supplementary Table 2.

### Reporting summary

Further information on research design is available in the Nature Research Reporting Summary linked to this article.

## Supplementary information

Supplementary Information

Description of Additional Supplementary Files

Supplementary Movie 1

Supplementary Movie 2

Supplementary Movie 3

Supplementary Movie 4

Supplementary Movie 5

Supplementary Data 1

Reporting Summary

## Data Availability

The authors declare that the data supporting the findings of this study are available within the paper (Supplementary Data 1). The data of the supplementary information are available from the corresponding author on reasonable request.
