# Supplementary material for: Squalenoyl siRNA PMP22 nanoparticles are effective in treating mouse models of Charcot-Marie-Tooth disease type 1 A
Source: Commun Biol. 2021 Mar 9;4:317. doi: 10.1038/s42003-021-01839-2 (PMC7943818; doi:10.1038/s42003-021-01839-2)
Supplement: Supplementary file 1 — Supplementary Information [file 42003_2021_1839_MOESM1_ESM.pdf]

Supplementary Information for the article

Squalenoyl siRNA PMP22 nanoparticles are effective in treating mouse models of Charcot-Marie-Tooth disease type 1A

Suzan Boutary[1], Marie Caillaud[1], Mévidette El Madani[1,2], Jean-Michel Vallat[3], Julien Loisel-Duwattez[1,4], Alice Rouyer[1], Laurence Richard[3], Céline Gracia[5], Giorgia Urbinati[5], Didier Desmaële[6], Andoni Echaniz-Laguna[1,4], David Adams[1,4], Patrick Couvreur[6], Michael Schumacher[1], Charbel Massaad[7] and Liliane Massaad-Massade[1*]

[1] U1195 Diseases and Hormones of the Nervous System, Inserm and University Paris-Saclay, 94276, Le Kremlin-Bicêtre, France.

[2] National Research Centre, Cairo, Egypt.

[3] Service de Neurologie - Centre de Référence Neuropathies Périphérique Rares, CHU de Limoges - Hôpital Dupuytren, 2 Avenue Martin Luther King, 87042 LIMOGES CEDEX, FRANCE

[4] Neurology Department, AP-HP, Université Paris-Saclay and French Reference Center for Familial Amyloid Polyneuropathy and other rare peripheral neuropathies (CRMR-NNERF), Bicêtre University Hospital, Le Kremlin-Bicêtre, France.

[5] UMR 8203 CNRS, newly UMR 9018 CNRS, Université Paris-Saclay, Villejuif, France 94805.

[6] Institut Galien Paris-Sud, CNRS UMR 8612, Université Paris-Sud, Université Paris-Saclay, 92290 Châtenay-Malabry, France.

[7] Faculty of Basic and Biomedical Sciences, Paris Descartes University, INSERM UMRS 1124, 75006 Paris, France.

***Corresponding author**: Liliane Massade, PhD, Inserm U1195, 94276 Le Kremlin-Bicêtre, France. Email: liliane.massade@inserm.fr, Phone : + 33 1 49 59 18 30

Supplementary Figures

Supplementary Figure 1

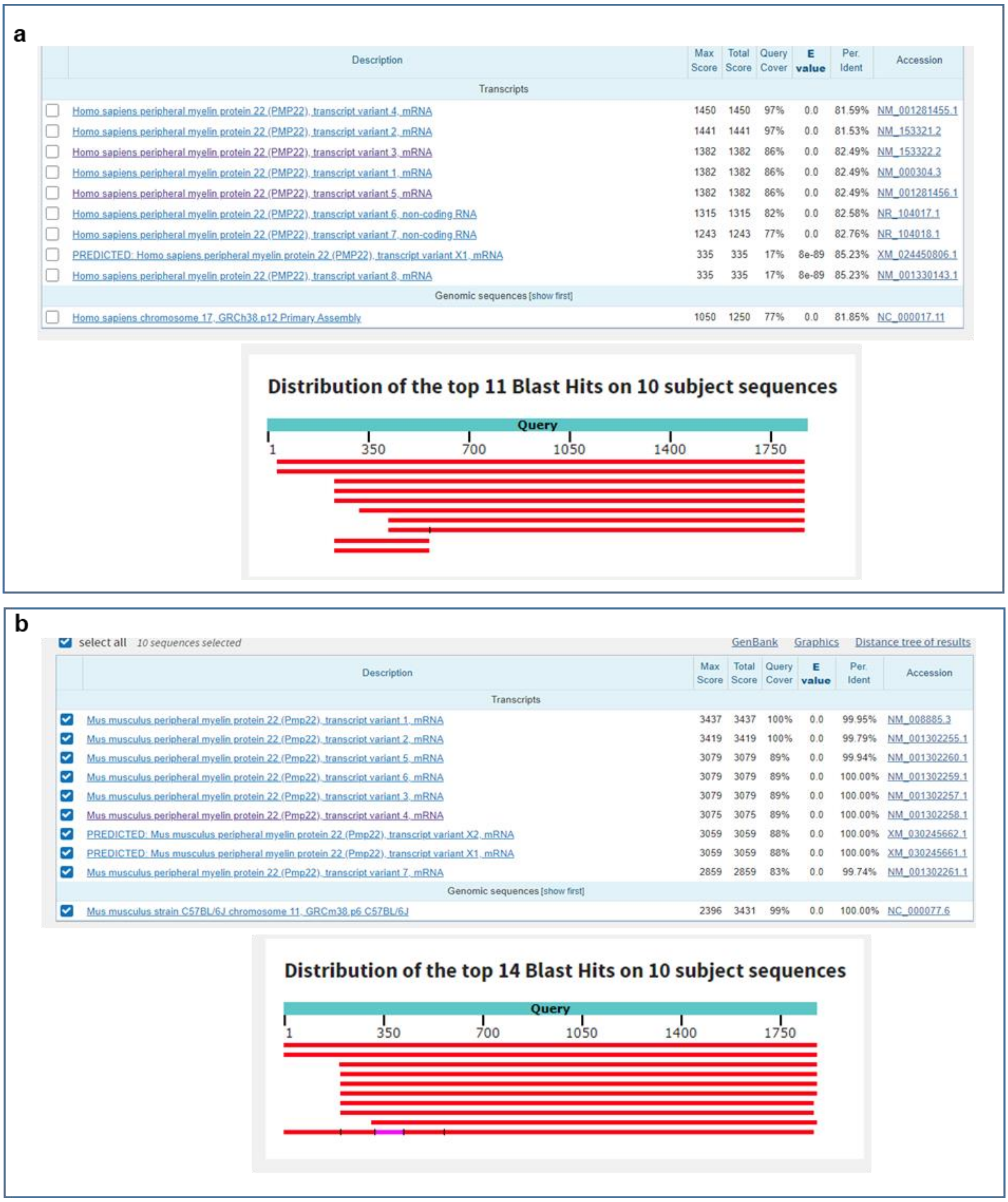

Supplementary Figure 1 | The common PMP22 mRNA sequence and homology between the variants of homosapiens and mus musculus. Using nucleotide blast, the PMP22 mRNA sequence was blasted to determine the homology between (**a**) *homo sapiens* and (**b**) *mus musculus* species. This common sequence recognizes all mRNA variants for both species.

Supplementary Figure 2

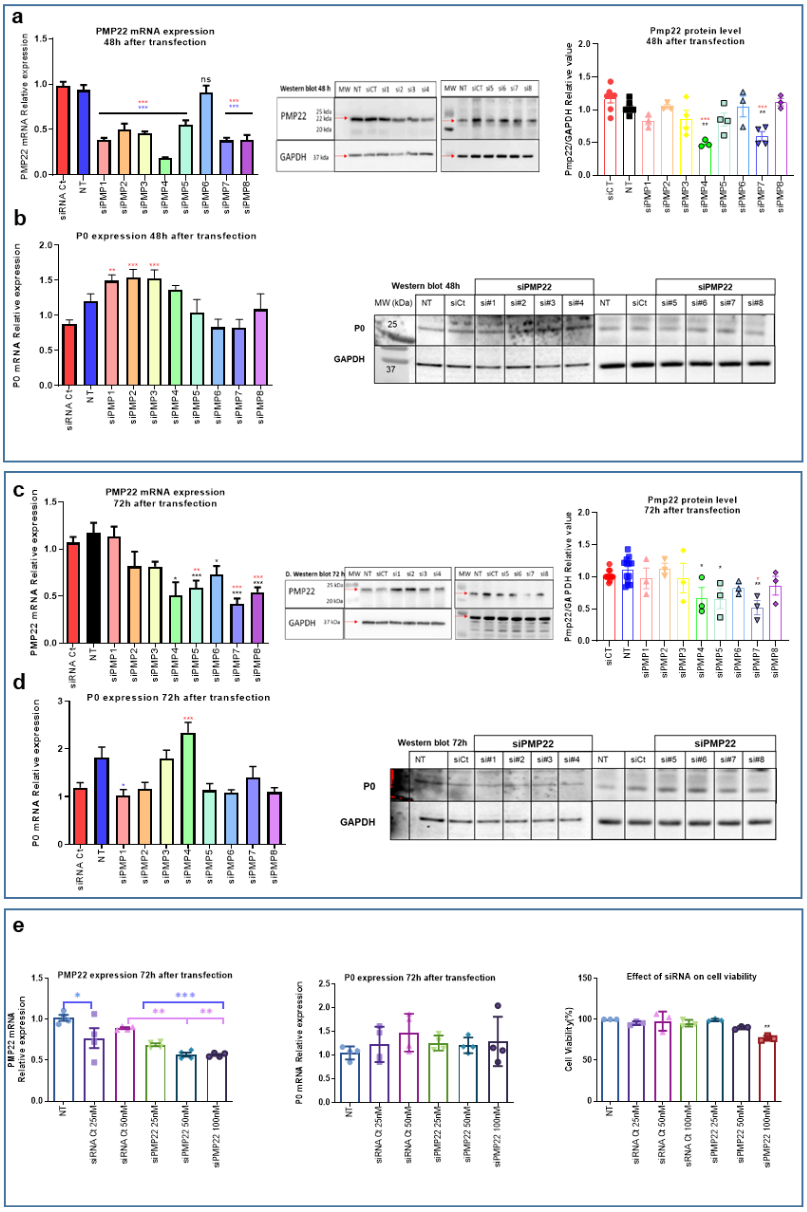

Supplementary Figure 2 | siRNA PMP7 inhibited PMP22 mRNA levels by 50% and had no effect on P0 levels and cell viability. a-d, eight different siRNAs PMP22 were transfected in MSC-80 cells at 50 nM concentration. **a,** at 48h, all siRNA PMP22 significantly inhibited siPMP22 gene expression except siRNA PMP22 N° 6. Western blot showed that only two siRNA PMP22 (4 and 7) inhibited around 50% Pmp22 protein level. **b,** at 48h a significant increase in P0 mRNA expression was shown by siRNA PMP22 N° 1, 2 and 3 which was not reflected on P0 protein levels. **c,** at 72h siRNA PMP22 N° 4-8 showed a long-lasting effect inhibition on PMP22 gene expression which was reflected by a decrease of Pmp22 protein level for three of them (N° 4, 5 and 7). Only siRNA PMP22 N°7, showed a 50% decrease of the protein content. **d,** at 72h two siRNA PMP22 (N° 1 and 4) showed an effect on P0 mRNA expression yet this was not reflected on the protein level. These results showed that N°7 (named siRNA PMP22) met all criteria (inhibition in a long-lasting manner by about 50% of PMP22). Therefore, different concentrations of siRNA PMP22 were used to investigate the effect on PMP22 and P0 mRNA expression, as well as, on cell viability **(e).** Concerning mRNA expression, all the tested concentrations of siRNA PMP22 inhibited PMP22, noteworthy siRNA Ct 25 nM affected PMP22 expression in the same manner. The tested concentrations did not affect P0 mRNA expression. Nevertheless, at 100 nM of siRNA PMP22, a significant decrease in cell viability was observed. Data represent mean ± s.e.m of at least three experiments (*: $p < 0.05$**: $p < 0.01$; ***: $p < 0.001$) when compared by ANOVA followed by Tukey's multiple comparison. Red and purple stars showed significance between siRNAs PMP22 and siRNA Ct, blue ones (dark and light) between NT cells and the dark star between all the groups

Supplementary Figure 3

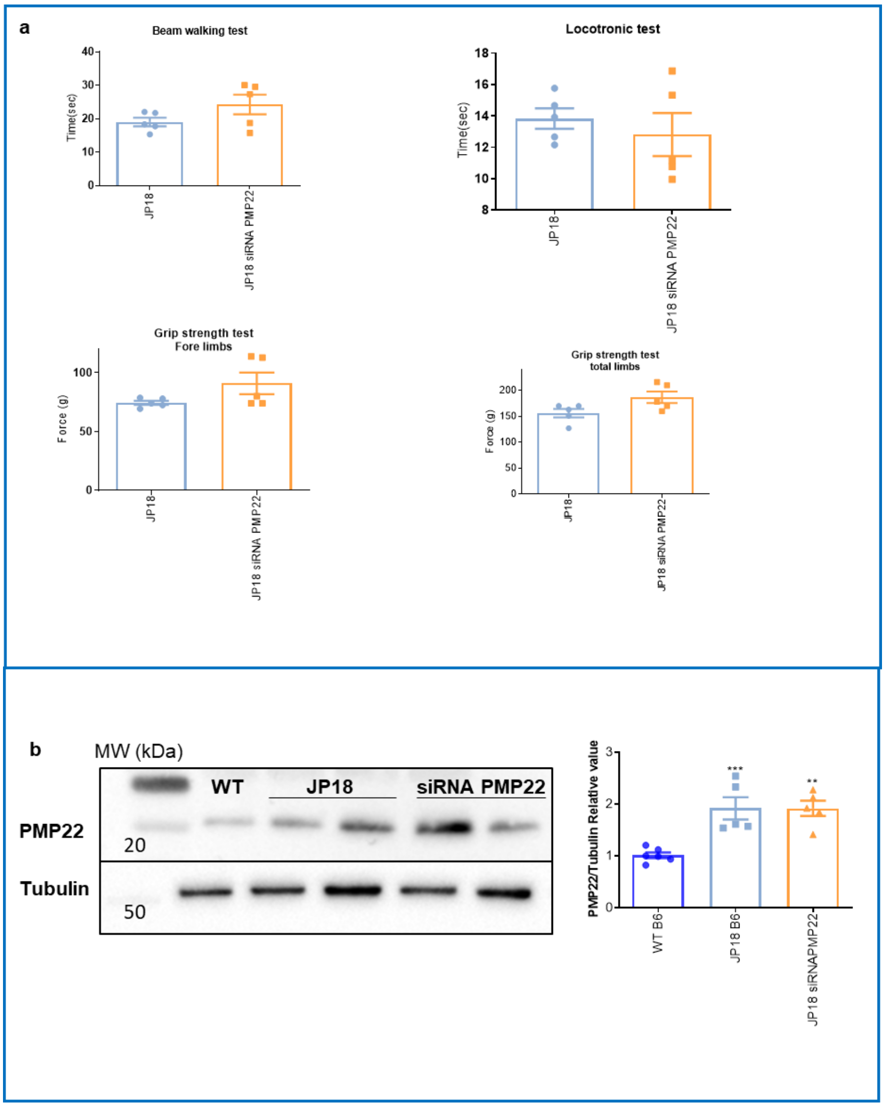

Supplementary Figure 3 | No effect was observed after treatment with naked siRNA PMP22 on JP18 CMT1A mice motor activity and Pmp22 protein level. a, analysis of behavioral tests: beam walking, locotronic and grip strength tests on JP18 mice treated with naked siRNA PMP22. No difference was detected between untreated and treated JP18 mice. Using Mann-Whitney test no statistical difference was observed between treated and untreated mice. **b,** a representative western blot of Pmp22 level. No effect of the treatment by siRNA PMP22 on Pmp22 protein level was observed after quantification by image J software. Data represent mean ± s.e.m (four mice per group). MW: molecular-weight size marker, *, $p < 0.05$, *** $p < 0.001$

Supplementary Figure 4

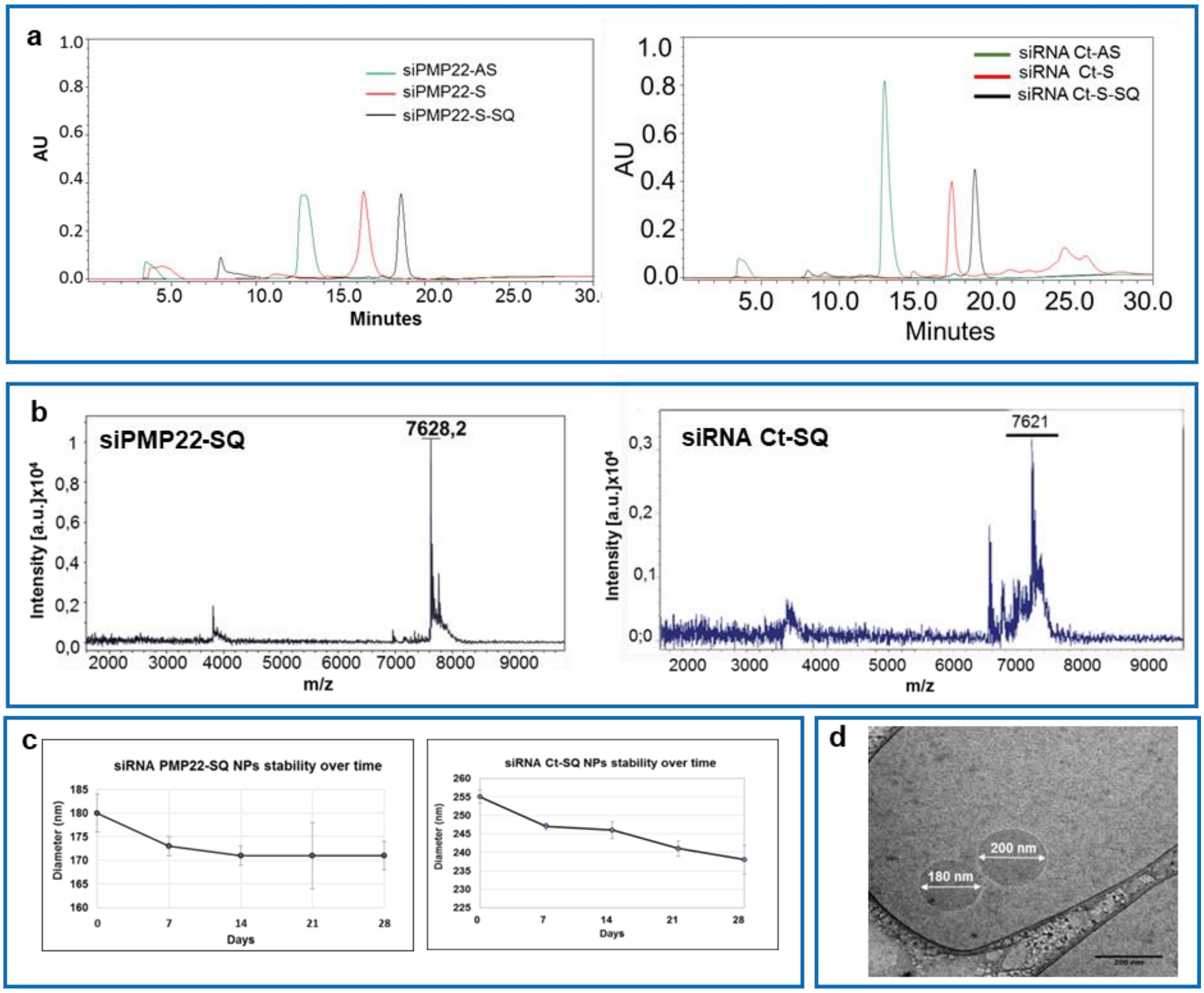

Supplementary Figure 4 | Characterization of siRNA PMP22-squalene (SQ) and siRNA Ct-SQ bioconjugates and nanoparticles. a, HPLC chromatograms of the siRNA PMP22 (left) and siRNA Ct (Right): antisense strand (AS) (green chromatogram), sense strand (S) (red chromatogram) and siRNA-SQ bioconjugates (black chromatogram). The elution time of siRNA-SQ bioconjugate was 18 mins for both siRNA bioconjugates and higher than that for both siRNA antisense and sense strand (13 and 15 mins, respectively). **b**, MALDI-TOF MS spectrum of siRNA PMP22-SQ and siRNA Ct-SQ bioconjugates showing a major peak with a molecular weight of 7628,2 Da for siRNA PMP22-SQ and of 7621 Da for siRNA Ct-SQ bioconjugates. **c**, Physicochemical characterization of siRNA PMP22-SQ and siRNA Ct-SQ NPs by dynamic light scattering (DLS). The size of both NPs was monitored over time (one month). Each measure represented the mean of 3 independent formulations and 3 independent technical measurements. **d**, CryoTEM image of the siRNA PMP22-SQ-NPs. White arrows indicate the nanoobject size detected (180 nm and 200 nm). The scale bar used is 200 nm.

Supplementary Figure 5

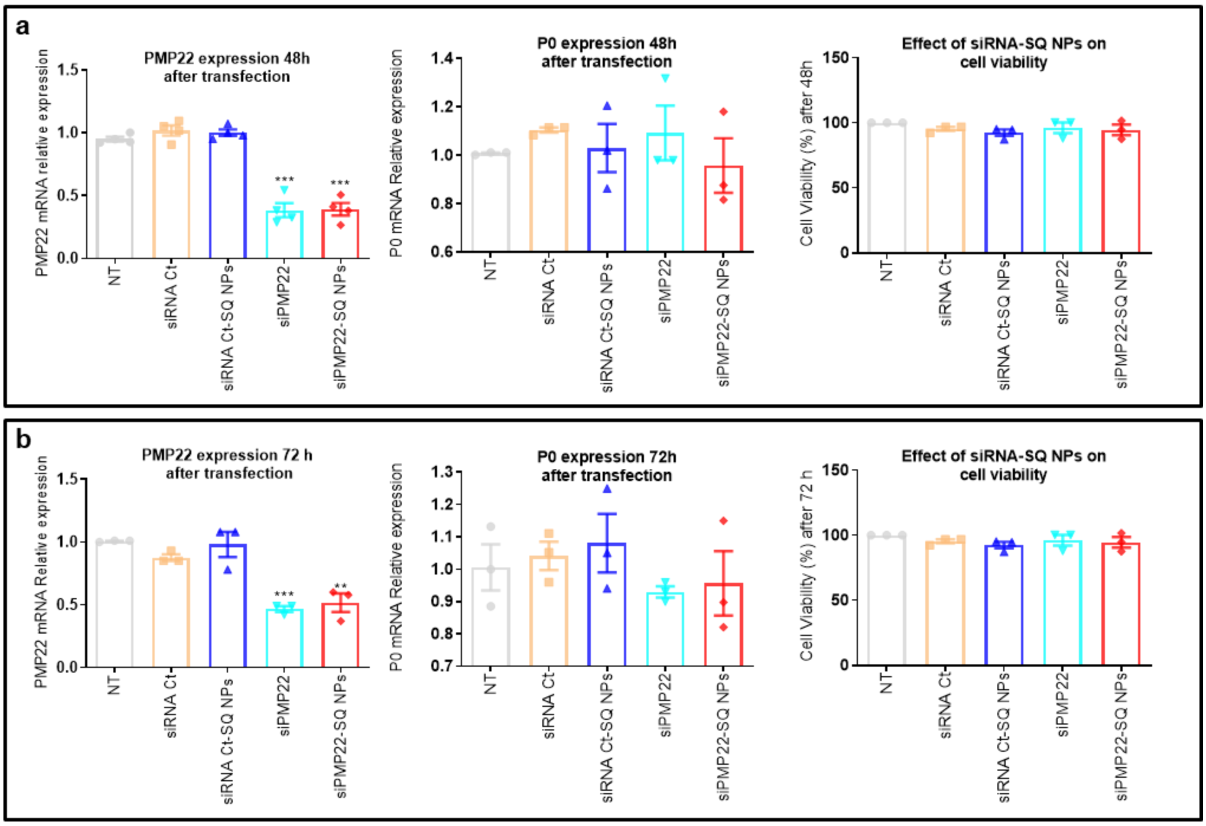

Supplementary Figure 5 | Chemical modifications did not affect the efficacy of siRNA PMP22 in MSC-80 cells. MSC80 cells were transfected with siRNA Ct, siRNA Ct-SQ NPs, siRNA PMP22 and siRNA PMP22-SQ NPs at a concentration of 50nM. After 48h and 72h, cells were harvested for either mRNA extraction to analyze gene knockdown or to perform MTT assay for cell viability. At 48h **(a)** and 72h **(b)**, the siRNA PMP22-SQ NPs significantly inhibited PMP22 mRNA expression by 50%, similarly to siRNA PMP22, and showed no significant effect neither on P0, nor on cell viability. siRNA Ct-SQ NPs behaved the same way as siRNA Ct, thus proving no effect of squalene and DBCO modification on the efficacy of siRNA PMP22. (*) represent significance between NT group and other groups, **, $p < 0.01$, *** $p < 0.001$ using Anova analysis followed by Tukey's multiple comparisons test. Each experiment is repeated at least 3 times.

Supplementary Figure 6

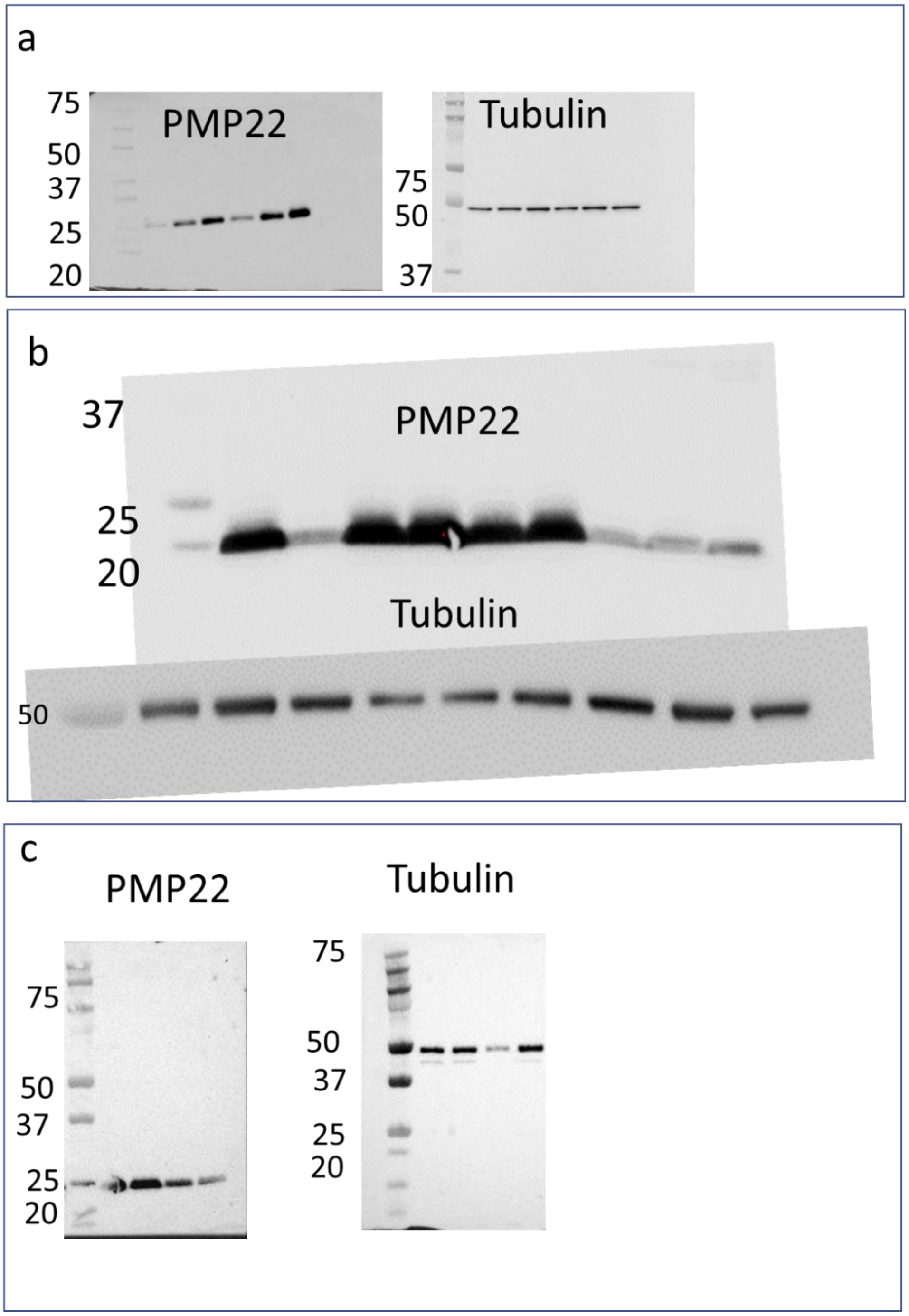

Supplementary Figure 6 | Uncropped blot/gel images. a corresponds to figure 1a, **b** corresponds to figure 3a and **c** corresponds to figure 3d.

Supplementary Figure 7

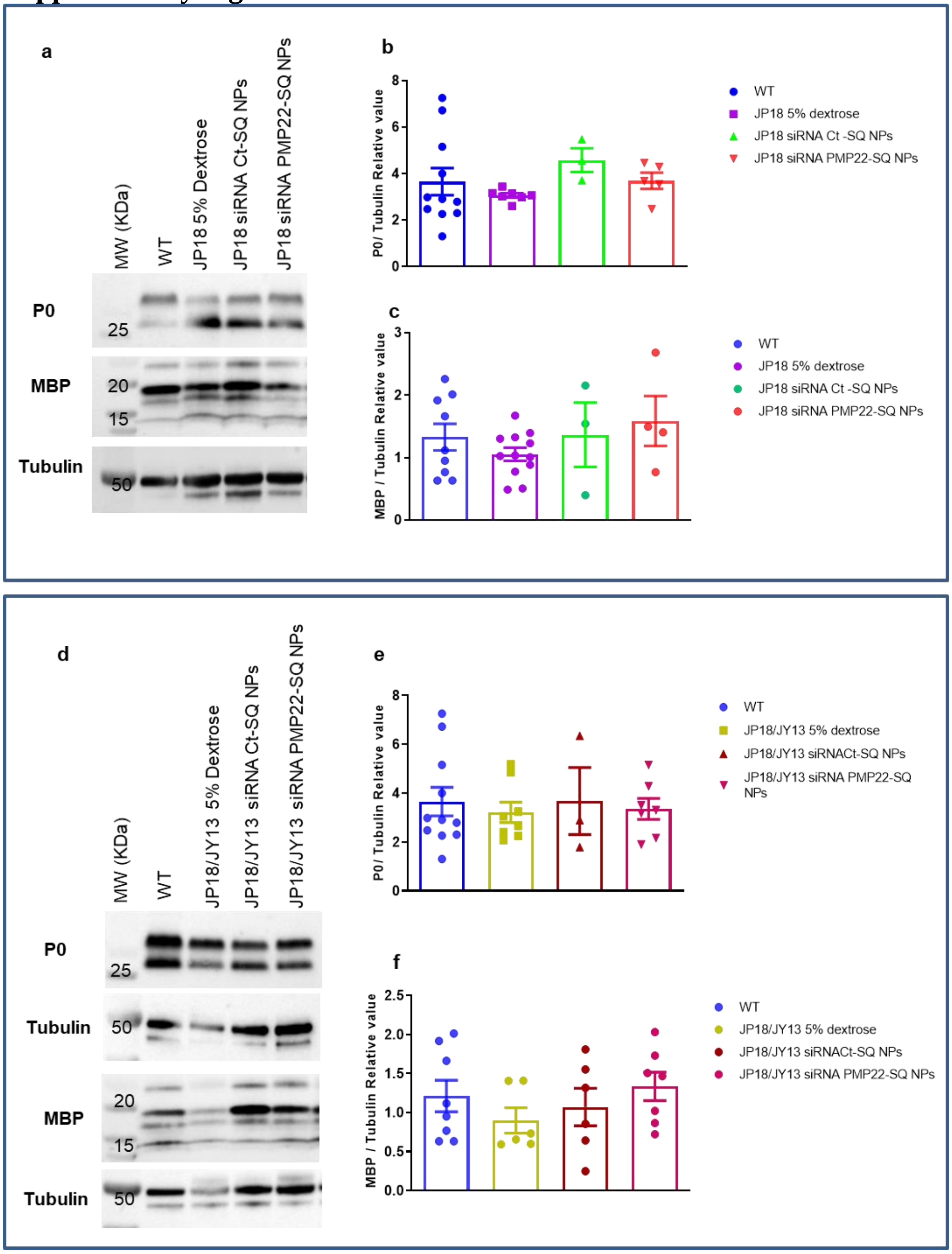

Supplementary Figure 7 | siRNA PMP22-SQ treatment has no effect on P0 and MBP proteins. a and d representative images of protein expression of P0 and MBP in JP18 and JP18/JY13 treatment groups respectively. **b, c, e & f** show quantification analysis of protein bands of P0 and MBP normalized over tubulin. Data represent mean ± s.e.m of 9 mice for JP18 and 6 mice for JP18/JY13. No significant effect of siRNA PMP22-SQ NPs treatment was reported on both P0 and MBP. Anova analysis followed by Tukey's multiple comparisons test was used.

Supplementary Figure 8

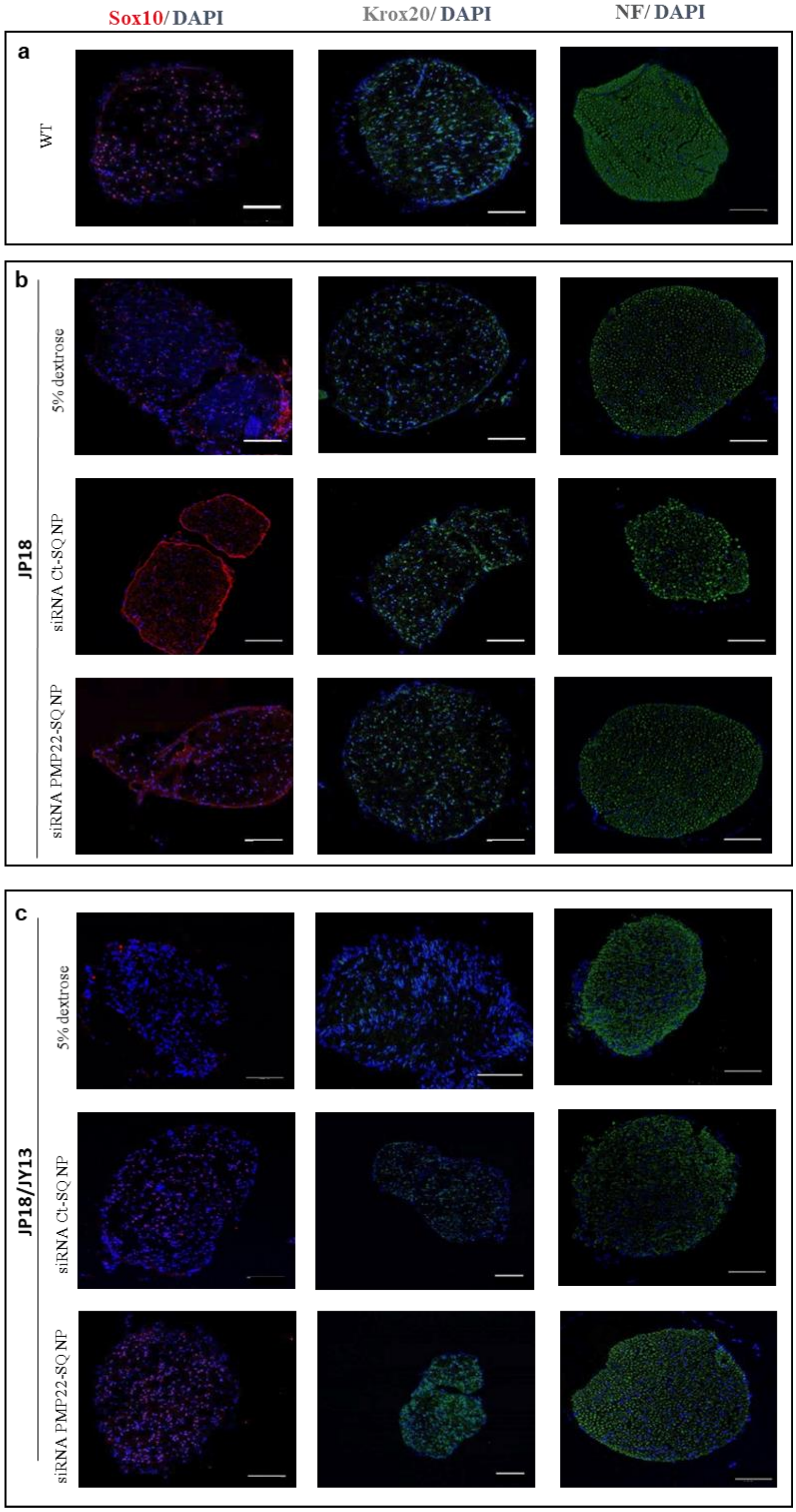

Supplementary Figure 8 | Normalization of Sox10, Krox20 and neurofilaments (NF) by siRNA PMP22-SQ NPs. Representative confocal microscopy images of immune-stained sciatic nerve sections of 5µm thickness showing Sox10 (red fluorescence), Krox 20 (green fluorescence) and NF (green fluorescence). **a,** shows images of WTB6 group. **b**, images of JP18 treatment groups and **c,** represents images of JP18/JY13 treatment groups. DAPI staining (blue) was used to colorize the nucleus. Magnification lens 20X. Scale bar 100 µm.

Supplementary Figure 9

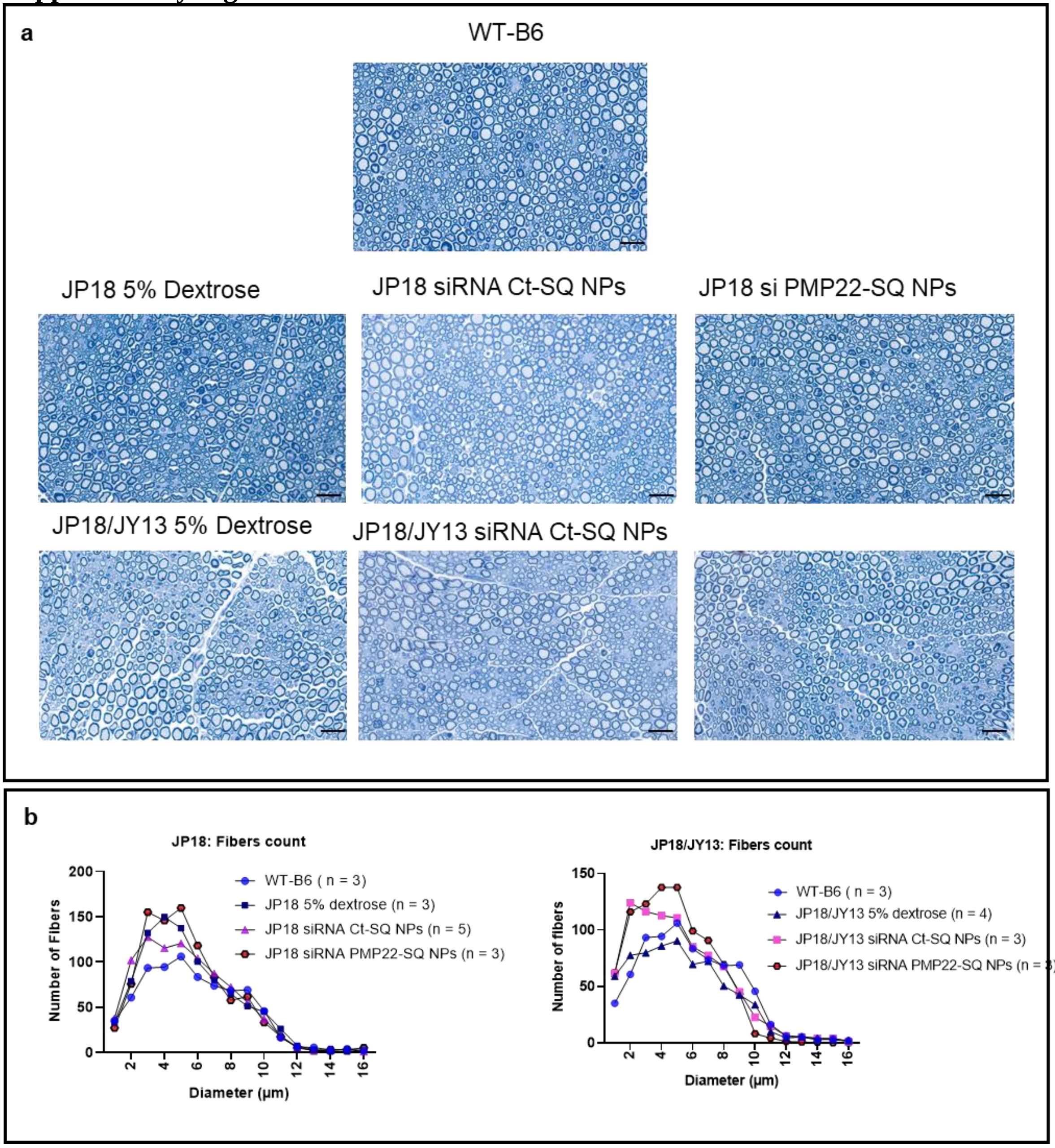

Supplementary Figure 9 | siRNA PMP22-SQ NPs did not affect fiber density in both CMT1A mouse models. a, Representative images of semi-thin section of sciatic nerves from WT, JP18 and JP18/JY13 mice treated with 5% dextrose, siRNA Ct-SQ NPs and siRNA PMP22-SQ NPs. Sciatic nerve sections were scanned at 40x and pictures were snapped at 70x for counting the number of myelinated fibers using image J software (scale bar 20 µm). **b**, Fiber count measure on JP18 and JP18/JY13 models. Using quadratic model analysis, no statistical difference was observed between the siRNA PMP-SQ NPs and the other groups for both the JP18 and JP18/JY13 mouse models.

Supplementary Figure 10

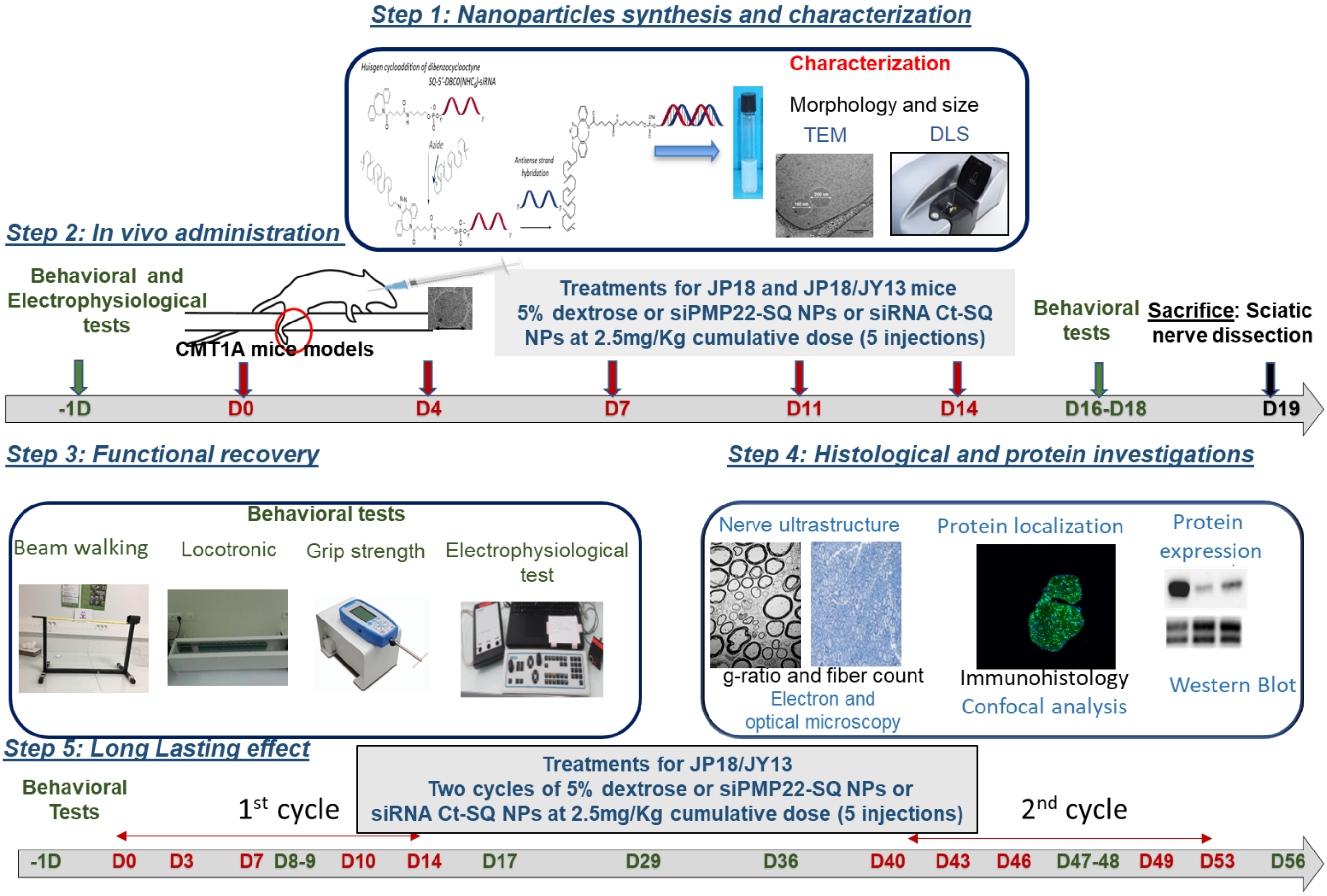

Supplementary Figure 10 | Detailed experimental protocol summarizing the steps of the study. Step 1, represents the siRNA-SQ NPs synthesis and characterization. Step 2, is the in vivo administration of siRNA-SQ NPs. Step 3 includes the functional recovery tests done in the study. Step 4, summarizes the histological and molecular tests performed. Step 5, represents the schedule of treatment for the long-lasting effect experiment where treatment was stopped at day 14 and then reinitiated at day 40.

Supplementary Tables

Supplementary Table 1: Design and position of siRNAs targeting PMP22.

a. Common mRNA PMP22 sequence between homosapiens and mus musculus

ACACCCTTCTGCAGCGACGCAAATAGGGCGTAGTTCCCGTTAAAGGGGAACACCGGGAGCCTCCCACTGCCCCCT
TGCTTTGCGCGCGCGCTGACCCGCAGCACAGCTGTCTTTGGGGACGCCAGCAACCCAGTGGACGCACCGGAGTTT
GTGCCGAGGCTAATCTGCTCTGAGATAGCTGTCCCTTTGAACTGAAACAGGCACCGCTCCTCTGATCCCGAGCCC
AACTCCCAGCCACC**ATG**CTCCTACTCTTGTTGGGGATCCTGTTCCTGCACATCGCGGTGCTAGTGTTGCTCTTCG
TCTCCACCATCGTCAGCCAATGGCTCGTGGGCAATGGACACACGACTGATCTCTGGCAGAACTGTACCACATCCG
CCTTGGGAGCCGTCCAACACTGCTACTCCTCATCAGTGAGCGAATGGCTGCAGTCTGTCCAGGCCACCATGATCC
TGTCTGTCATCTTCAGCGTCCTGGCTCTGTTCCTGTTCTTCTGCCAGCTCTTCACTCTCACCAAAGGCGGCCGGT
TTTACATCACTGGATTCTTCCAAATCCTTGCTGGTCTGTGCGTGATGAGTGCAGCGGCCATCTACACAGTGAGGC
ACAGTGAGTGGCATGTCAACACTGACTACTCCTATGGCTTCGCCTACATCCTGGCCTGGGTGGCCTTTCCCCTAG
CCCTCCTCAGTGGTATCATCTATGTGATCCTGCGGAAACGCGAATGAGGCGCCCGACGACGCACCGTCCGTCTAG
GCTCTGAGCGCGCATAGGGTCCACAGGGAGGGAGGAAGGAAACCAGAGAACAAAACCAACCAACCAAAAAAGAGC
TAGCCCCAAACCCAAACGCAAGCCAAACCAAACAGAACGCAGTTGAGTGGGGATTGCTGTTGATTGAAGATGTAT
ATAATATCTATGGTTTATAAA**ACCTATTTATAACACTTTT**TACATATATGTACATAGGATTGTTTTGCTTTTT
ATGTTGACCGTCAGCCTCGTGTTGAATCTTAAACAACTTTACATCCTAACACTATAACCAAGCTCAGTATCTTTG
TTTTGTTTCGTTTTTTTTTTTAATCTTTTTGTTTTGCTCAGACATAAAAACTCCACGTGGCCCCCTTTCATCTGA
AAGCAGATACCTCCCTCCCACTCAACCTCATAGGATAACCAAAGTGTGGGGACAAACCCCAGACAGTTGAAGACC
TTTACACTATGGGTGACCCAGTGCATTTAGCAGGAGTATCCACTGCCCGAATCCATGTGTGAAGCCCTAAGCACT
CACAGACGAAAAGCCCTGACCGGAACCCTCTGCAAAAACAGTAATAGCTGGTGGCTCCTGAACACTTGACCCTGT
AGACGGAGTACTGGGGCCACACGTTTAAATGAGAAGTCAGAGACAAGCAATCTGTGAAATGGTGCTATAGATTTA
CCATTCCTTGTTATTACTAATCGTTTAAACCACTCACTGGAAACTCAATTAACAGTTTTATGCGATACAGCAGAA
TGGAGACCCGATACAAACGGTTCATAACTGCTTTCATACCTAGCTAGGCTGTTGTTATTACTACAATAAATAAAT
CTCAAAGCCTTCGTCAGTCCCACAGTTTTCTCACGGTCGGAGCATCAGGACGAGCATCTAGACCCTTGGGACTAG
CGAGTTCCCTGGCTTTCTGGGTCTAGAGTGTTCTGTGCCTCCAAGGACTGTCTGGCGATGACTTGTATTGGCCAC
CAACTGTAGATGTATATACGGTGTCCTTCTGATGCTAAGACTCCAGACCTTTCTTGTTTTTGCTTGCTTTCTCTG
ATTTTATACCAACTGTGTGGACTAAGATGCATCAAAATAAACATCAGAGTAACTCAAAAAAAAAAA

b. sequence of siRNAs

LAB NUMBER	POSITION WITHIN THE SEQUENCE	SEQUENCE	Total score
1	siPMP22 473-492	GGCUCUGUUCCUGUUCUUC[dT][dT]	3
	siPMP22 473-492_as	GAAGAACAGGAACAGAGCC[dT][dT]	
2	siPMP22 922-941	ACCUAUUUAUAACACUUUU[dT][dT]	6
	siPMP22 922-941_as	AAAAGUGUUAUAAAUAGGU[dT][dT]	
3	siPMP22 1561-1580	ACAAUAAAUAAAUCUCAAA[dT][dT]	5
	siPMP22 1561-1580_as	UUUGAGAUUUAUUUAUUGU[dT][dT]	
4	siPMP22 988-1007	CCUCGUGUUGAAUCUUAAA[dT][dT]	8
	siPMP22 988-1007_as	UUUAAGAUUCAACACGAGG[dT][dT]	
5	siPMP22 1720-1739	CCACCAACUGUAGAUGUAU[dT][dT]	5
	siPMP22 1720-1739_as	AUACAUCUACAGUUGGUGG[dT][dT]	
6	siPMP22 430-449	CUGUCCAGGCCACCAUGAU[dT][dT]	4
	siPMP22 430-449_as	AUCAUGGUGGCCUGGACAG[dT][dT]	
7	siPMP22 1806-1825	AUACCAACUGUGUGGACUA[dT][dT]	7
	siPMP22 1806-1825_as	UAGUCCACACAGUUGGUAU[dT][dT]	
8	siPMP22 921-940	AAACCUAUUUAUAACACUU[dT][dT]	5
	siPMP22 921-940_as	AAGUGUUAUAAAUAGGUUU[dT][dT]	

Supplementary Table 1 | Design and position of siRNAs targeting PMP22. a, Position of siRNAs PMP22 in the common PMP22 mRNA sequence among *homo sapiens* and *mus musculus*. Highlighted: Position of designed siRNA within the sequence, in bold N°2 sequence, underlined, sequence N°8. **b,** The Table shows the sequences of 8 different siRNAs and their corresponding score calculated according "Reynolds scores". Highly efficient siRNA should have a score ≥ 6

Supplementary Table 2: Formulas of the quadratic equations used to calculate the Cut-off

WT B6: Fibre count = (0.9389+33.6059*log(Diameter) -28.7654*log(Diameter)*log(Diameter))^2

JP18/JY13 5% dextrose: Fibre count = (3.5711+23.9332*log(Diameter) -22.3691*log(Diameter)*log(Diameter))^2
JP18/JY13 siRNA Ct-SQ NPs: Fibre count = (5.5638+22.0609*log(Diameter) -22.3691*log(Diameter)*log(Diameter))^2
JP18/JY13 siRNA PMP22-SQ NPs: Fibre count = (6.5679+20.4019*log(Diameter) -22.3691*log(Diameter)*log(Diameter))^2

JP18 5% dextrose: Fibre count = (2.4599+32.1679*log(Diameter) -28.7654*log(Diameter)*log(Diameter))^2
JP18 siRNA Ct-SQ NPs: Fibre count = (2.7245+31.7374*log(Diameter) -28.7654*log(Diameter)*log(Diameter))^2
JP18 siRNA PMP22-SQ NPs: Fibre count = (2.4911+32.2806*log(Diameter) -28.7654*log(Diameter)*log(Diameter))^2

Supplementary Table 3: Heart and kidney weights over body weight ratio and biochemical blood analysis of JP18 and JP18/JY13 treatment groups.

**Mice groups**	**B.W. (g) ± SD**	**H.W./B.W. ± SD**	**K.W/B.W ± SD**	**Albumin (g/L) ± SD**	**Creatinine (µmol/L) ± SD**	**Cholesterol (mmol/L) ± SD**	**LDL-C (mmol/L) ± SD**	**HDL-C (mmol/L) ± SD**	**AST (UI/L) ± SD**	**ALT (UI/L) ± SD**
**WT**	24.8 ± 2.6	0.48 ± 0.05	1.11 ± 0.16	31.8 ± 0.56	11 ± 7	1.64 ± 0.28	0.2 ± 0.06	1.36 ± 0.35	ND	ND
**JP18 5% dextrose**	25.6 ± 1.1	0.46 ± 0.07	1.09 ± 0.17	31.2 ± 2.28	11.7 ±0.6	1.67 ± 0.23	0.23 ±0.04	1.45 ±0.22	ND	ND
**JP18 siPMP22-SQ NPs**	26.4 ± 2.1	0.48 ± 0.13	1.22 ± 0.14	32.6 ± 0.85	9.8 ± 0.95	1.64 ± 0.13	0.23 ± 0.03	1.43 ±0.12	ND	ND
**JP18/JY13 5% dextrose**	22.8 ± 3.7	0.52 ± 0.11	1.30 ± 0.15	31.4 ± 2.0	10.2 ± 1.7	1.60 ± 0.19	0.21 ± 0.05	1.3 ± 0.33	109 ± 26	27 ± 9
**JP18/JY13 siRNA Ct-SQ NP**	21.7 ± 2.5	0.46 ± 0.04	1.27 ± 0.12	32.5 ± 2.6	14 ± 4.4	1.55 ± 0.23	0.24 ± 0.04	1.21 ± 0.19	136 ± 69	27 ± 20
**JP18/JY13 siPMP22-SQ NPs**	21.1 ± 1.5	0.59 ± 0.09	1.35 ± 0.16	32.4 ± 1.53	12.2 ± 3.5	1.28 ± 0.42	0.28 ± 0.04	0.95 ± 0.39	105 ± 30	25.5 ± 13

***B.W.** Body weight**, H.W.** Heart weight, **K.W.** Kidney weight, **AST** Aspartate Transaminase**, ALT** Alanine Amino transferase. **ND =** not dosed. **Note:** The table lacks the measure on the plasma liver enzymatic activity due to low volume of blood samples from the JP18 mice but it was performed on the JP18/JY13 mice after two cycles of treatment. For all the parameters tested, no statistical difference was found between WT and treatments and in between the different treated groups.

Supplementary Table 4: Primary and secondary antibodies of IHC protocol

Primary Antibodies	Dilution	Secondary Antibodies	Dilution
**Erg**2**/Krox20 Rabbit polyclonal** (Thermo Fischer Scientific**,** PA5-27814**)**	1/500	Goat anti-rabbit IgG /Alexa Fluor 488 conjugate (Invitrogen, A32731)	1/500
**SOX10 Goat polyclonal (** R&D systems, AF2864**)**	1/100	Donkey anti-goat IgG/CY3-conjugated (Jackson Immuno Research, AP194C)	1/500
**Anti-Neurofilament Rabbit polyclonal (**EMD Millipore, AB1989**)**	1/1000	Goat anti-rabbit IgG /Alexa Fluor 488 conjugate (Invitrogen, A32731)	1/1000
