# Supplementary material for: Squalenoyl siRNA PMP22 nanoparticles are effective in treating mouse models of Charcot-Marie-Tooth disease type 1 A
Source: Commun Biol. 2021 Mar 9;4:317. doi: 10.1038/s42003-021-01839-2 (PMC7943818; doi:10.1038/s42003-021-01839-2)
Supplement: Supplementary file 2 — Description of Additional Supplementary Files [file 42003_2021_1839_MOESM2_ESM.pdf]

Description of Additional Supplementary Files

Supplementary Video 1: Effect of naked siRNA PMP22 on JP18 mice. Video shows the time taken by JP18 mice before and after treatment with 2.5mg of naked siRNA PMP22 to cross the bar of the beam walking test.

Supplementary Video 2: Comparison between the mice strains. Video shows the time taken by WT B6, JP18 and JP18/JY13 mice to cross the bar of the beam walking test.

Supplementary Video 3: Effect of siRNA PMP22-SQ NPs on JP18 mice. Video shows the time taken by WT B6, JP18 and JP18 mice treated with siRNA PMP22-SQ NPs to cross the bar of the beam walking test.

Supplementary Video 4: Effect of siRNA PMP22-SQ NPs on JP18/JY13 mice. Video shows the time taken by a JP18/JY13 mouse before and after treatment with siRNA PMP22-SQ NPs to cross the bar of the beam walking test.

Supplementary Video 5: Effect of siRNA PMP22-SQ NPs on highly affected mice. Video shows the behavior of a highly affected JP18/JY13 mouse before and after treatment with siRNA PMP22-SQ NPs.

Supplementary Data 1: Source data for all graphs.
