# Supplementary material for: Squalenoyl siRNA PMP22 nanoparticles are effective in treating mouse models of Charcot-Marie-Tooth disease type 1 A
Source: Commun Biol. 2021 Mar 9;4:317. doi: 10.1038/s42003-021-01839-2 (PMC7943818; doi:10.1038/s42003-021-01839-2)
Supplement: Supplementary file 9 — Reporting Summary [file 42003_2021_1839_MOESM9_ESM.pdf]

nature research

Corresponding author(s): Dr. Liliane Massaad-Massade

Last updated by author(s): Feb 1, 2021

Reporting Summary

Nature Research wishes to improve the reproducibility of the work that we publish. This form provides structure for consistency and transparency in reporting. For further information on Nature Research policies, see our Editorial Policies and the Editorial Policy Checklist.

Statistics

For all statistical analyses, confirm that the following items are present in the figure legend, table legend, main text, or Methods section.

n/a	Confirmed	
☐	☒	The exact sample size (n) for each experimental group/condition, given as a discrete number and unit of measurement
☐	☒	A statement on whether measurements were taken from distinct samples or whether the same sample was measured repeatedly
☐	☒	The statistical test(s) used AND whether they are one- or two-sided<br>*Only common tests should be described solely by name; describe more complex techniques in the Methods section.*
☐	☒	A description of all covariates tested
☐	☒	A description of any assumptions or corrections, such as tests of normality and adjustment for multiple comparisons
☐	☒	A full description of the statistical parameters including central tendency (e.g. means) or other basic estimates (e.g. regression coefficient) AND variation (e.g. standard deviation) or associated estimates of uncertainty (e.g. confidence intervals)
☐	☒	For null hypothesis testing, the test statistic (e.g. F, t, r) with confidence intervals, effect sizes, degrees of freedom and P value noted<br>*Give P values as exact values whenever suitable.*
☒	☐	For Bayesian analysis, information on the choice of priors and Markov chain Monte Carlo settings
☒	☐	For hierarchical and complex designs, identification of the appropriate level for tests and full reporting of outcomes
☒	☐	Estimates of effect sizes (e.g. Cohen's d, Pearson's r), indicating how they were calculated

Our web collection on statistics for biologists contains articles on many of the points above.

Software and code

Policy information about availability of computer code

Data collection	No software was used
Data analysis	image J software, GraphPad prism 8

For manuscripts utilizing custom algorithms or software that are central to the research but not yet described in published literature, software must be made available to editors and reviewers. We strongly encourage code deposition in a community repository (e.g. GitHub). See the Nature Research guidelines for submitting code & software for further information.

Data

Policy information about availability of data

All manuscripts must include a data availability statement. This statement should provide the following information, where applicable:

- Accession codes, unique identifiers, or web links for publicly available datasets
- A list of figures that have associated raw data
- A description of any restrictions on data availability

The authors declare that the data supporting the findings of this study are available within the paper (Supplementary Data1). The data of the supplementary information are available from the corresponding author on reasonable request.
Main figures 1 to 6 has associated raw data found in Supplementary Data 1

Field-specific reporting

Please select the one below that is the best fit for your research. If you are not sure, read the appropriate sections before making your selection.

- [x] Life sciences
- [ ] Behavioural & social sciences
- [ ] Ecological, evolutionary & environmental sciences

For a reference copy of the document with all sections, see nature.com/documents/nr-reporting-summary-flat.pdf

Life sciences study design

All studies must disclose on these points even when the disclosure is negative.

Sample size	For the invitro experiments all the experiments were done at least three times and repeated in duplicates.<br>for the JP18 mice treatment the number of mice is 9 per group<br>for the JP18/JY13 mice treatment: the sample size is 6<br>for the long lasting effect experiment the number of animals is 6 per group
Data exclusions	some data were excluded by calculating the outliers using the following link:https://www.graphpad.com/quickcalcs/Grubbs1.cfm
Replication	The Treatment on JP18 was repeated twice<br>All th invitro experiment were repeated at least three times with biological duplicates
Randomization	The mice were randomly allocated to each treatment group
Blinding	The investigators were blindly allocated to data collection and analysis

Reporting for specific materials, systems and methods

We require information from authors about some types of materials, experimental systems and methods used in many studies. Here, indicate whether each material, system or method listed is relevant to your study. If you are not sure if a list item applies to your research, read the appropriate section before selecting a response.

Materials & experimental systems

n/a	Involved in the study	
[ ]	[x]	Antibodies
[ ]	[x]	Eukaryotic cell lines
[x]	[ ]	Palaeontology and archaeology
[ ]	[x]	Animals and other organisms
[x]	[ ]	Human research participants
[x]	[ ]	Clinical data
[x]	[ ]	Dual use research of concern

Methods

n/a	Involved in the study	
[x]	[ ]	ChIP-seq
[x]	[ ]	Flow cytometry
[x]	[ ]	MRI-based neuroimaging

Antibodies

Antibodies used	anti PMP22 antibody, Sigma, SAB4502217<br>anti-myelin protein zero (P0),abcam ab31851<br>anti-MBP antibody, Abcam: ab7349<br>monoclonal GAPDH-HRP linked ,Cell Signalling technology, Ref: 3683<br>monoclonal anti tubulin ,Cell signalling, mAb#3873<br>Erg2/Krox20 Rabbit polyclonal, Thermo Fischer Scientific, PA5-27814<br>SOX10 Goat polyclonal, R&D systems, AF2864<br>Anti-Neurofilament Rabbit polyclonal ,EMD Millipore, AB1989
Validation	anti PMP22, Sigma, SAB4502217 (Applications: western blotting and reacts with mouse, Reference: Caillaud M, Chantemargue B, Richard L, et al. Local low dose curcumin treatment improves functional recovery and remyelination in a rat model of sciatic nerve crush through inhibition of oxidative stress. Neuropharmacology. 2018;139:98-116. doi:10.1016/j.neuropharm.2018.07.001)<br>anti-myelin protein zero (P0),abcam ab31851 (Applications: WB and react with mouse, rat and human samples. Reference: Chen G, Luo X, Wang W, Wang Y, Zhu F, Wang W. Interleukin-1β Promotes Schwann Cells De-Differentiation in Wallerian Degeneration via the c-JUN/AP-1 Pathway. Front Cell Neurosci. 2019;13:304. Published 2019 Jul 9. doi:10.3389/fncel.2019.00304)<br>Anti-Myelin Basic Protein antibody [12] ab7349 (Applications: western Blot)<br>monoclonal GAPDH-HRP linked ,Cell Signalling technology, Ref: 3683 (Specificity / Sensitivity, GAPDH (14C10) Rabbit mAb (HRP Conjugate) detects endogenous levels of total GAPDH protein.Species Reactivity: Human, Mouse, Rat, Monkey, Bovine, Pig.Application Key: W-Western IP-Immunoprecipitation IHC-Immunohistochemistry ChIP-Chromatin Immunoprecipitation IF-Immunofluorescence F-Flow Cytometry E-P-ELISA-Peptide.https://www.cellsignal.com/products/antibody-conjugates/gapdh-14c10-

rabbit-mab-hrp-conjugate/3683)
monoclonal anti tubulin ,Cell signalling, mAb#3873 (Application Western blot, Reactivity: mouse, rat and human. https://www.cellsignal.com/products/primary-antibodies/a-tubulin-dm1a-mouse-mab/3873)
Erg2/Krox20 Rabbit polyclonal, Thermo Fischer Scientific, PA5-27814 (Application: Immunohistochemistry (Frozen) (IHC (F)) and reactivity mouse and human. https://www.thermofisher.com/antibody/product/EGR2-Antibody-Polyclonal/PA5-27814)
SOX10 Goat polyclonal, R&D systems, AF2864 (Application: Immunocytochemistry .https://www.rndsystems.com/products/human-sox10-antibody_af2864)
Anti-Neurofilament Rabbit polyclonal ,EMD Millipore, AB1989 (Application: Immunohistochemistry (Frozen) (IHC (F)) and reactivity: mouse. https://www.labome.com/product/EMD-Millipore/AB1989.html)

Eukaryotic cell lines

Policy information about cell lines

Cell line source(s)	Dr. Charbel Massaad Laboratory, Paris Descartes University, Paris, France
Authentication	N/A
Mycoplasma contamination	Cell lines tested negative for mycoplasma contamination
Commonly misidentified lines (See ICLAC register)	N/A

Animals and other organisms

Policy information about studies involving animals; ARRIVE guidelines recommended for reporting animal research

Laboratory animals	JP18 transgenic CMT1A mouse model, age:16 weeks and gender used both males and female<br>JY13 transgenic CMT1A mouse model, used for breeding of JP18/JY13 CMT1A transgenic mouse model<br>JP18/JY13 transgenic CMT1A mouse model used at age of 12 weeks and gender used both males and females
Wild animals	The study did not contain animal captured from the field
Field-collected samples	The study didnot involve samples collected from the field
Ethics oversight	All animal experiments were approved by the institutional Ethics Committee of Animal Experimentation (CEEA) and research council, registered in the French Ministry of Higher Education and Research « Ministère de l'Enseignement Supérieur et de la Recherche; MESR, autorisation N°: APAFIS#10131-2016112916404689 ». It carried out according to French laws and regulations under the conditions established by the European Community (Directive 2010/63/UE). Investigation has been conducted in accordance with the ethical standards and according to the Declaration of Helsinki. All efforts were made to minimize animal suffering. Administration of treatments was performed under isoflurane anesthesia and animals were sacrificed by cervical dislocation. All animals were housed in sterilised laminar flow caging system. Food, water and bedding were sterilized before animals were placed in the cages. Food and water were given ad libitum.

Note that full information on the approval of the study protocol must also be provided in the manuscript.
